# A Receptor Model With Binding Affinity, Activation Efficacy, and Signal Amplification Parameters for Complex Fractional Response Versus Occupancy Data

**DOI:** 10.3389/fphar.2019.00605

**Published:** 2019-06-11

**Authors:** Peter Buchwald

**Affiliations:** Department of Molecular and Cellular Pharmacology, Diabetes Research Institute, Miller School of Medicine, University of Miami, Miami, FL, United States

**Keywords:** affinity, biased agonism, constitutive activity, efficacy, free energy, G-protein–coupled receptors, ligand binding, partial agonism

## Abstract

In quantitative pharmacology, multi-parameter receptor models are needed to account for the complex nonlinear relationship between fractional occupancy and response that can occur due to the intermixing of the effects of partial receptor activation and post-receptor signal amplification. Here, a general two-state receptor model and corresponding quantitative forms are proposed that unify three distinct processes, each characterized with its own parameter: 1) receptor binding, characterized by *K*_d_, the equilibrium dissociation constant used for binding affinity; 2) receptor activation, characterized by an (intrinsic) efficacy parameter *ε*; and 3) post-activation signal transduction (amplification), characterized by a gain parameter *γ*. Constitutive activity is accommodated via an additional *ε*_R0_ parameter quantifying the activation of the ligand-free receptor. Receptors can be active or inactive in both their ligand-free and ligand-bound states (two-state receptor theory), but ligand binding alters the likelihood of activation (induced fit). Because structural data now confirm that for most receptors in their active conformation, the small-molecule ligand-binding site is buried inside, straightforward binding to the active form (direct conformational selection) is unlikely. The proposed general equation has parameters that are more intuitive and better suited for optimization by nonlinear regression than those of the operational (Black and Leff) or del Castillo–Katz model. The model provides a unified framework for fitting complex data including a) fractional responses that do not match independently measured fractional occupancies, b) responses measured after partial irreversible inactivation of the “receptor reserve” (Furchgott method), c) fractional responses that are different along distinct downstream pathways (biased agonism), and d) responses with constitutive receptor activity. Furthermore, unlike previous models, the present one can be reduced back for special cases of its parameters to consecutively nested simplified forms that can be used on their own when adequate (e.g., *ε*_R0_ = 0, no constitutive activity; *γ* = 1: *E*_max_ model for partial agonism; *ε* = 1: Clark equation).

## Introduction

The receptor concept, which is about a century old and undeniably represents “pharmacology’s big idea” (Rang, 2006), forms the basis of our current mechanism of drug action theories (Maehle et al., 2002); detailed reviews can be found in Neubig et al. (2003), Colquhoun (2006), Kenakin (2008), Jenkinson (2010), Ehlert (2015a), and Kenakin (2018b). Most commonly accepted receptor models are two-state models in which receptor occupancy and activation do not fully correspond (Katzung and Trevor, 2014; Rang et al., 2015). This is needed to describe well-recognized phenomena, such as partial agonism (partial activation despite full receptor occupancy) and the existence of receptor reserve (maximum or close to maximum activation at only partial occupancy) (Colquhoun, 2006; Kenakin, 2008; Kenakin, 2018b; Jenkinson, 2010). The quantitative forms of these models use one parameter to characterize the binding affinity of the ligand (typically *K*_d_) and another one to characterize efficacy (e.g., *τ*, *ε*, *K_ε_*). However, outside of a core of quantitative pharmacology experts, these forms, including those of the operational (Black and Leff) and the minimal two-state (del Castillo–Katz) model, are not widely used because their parameters are not very intuitive, cumbersome to interpret, and often difficult to fit, plus they cannot be reduced to reproduce more widely used simple models (e.g., Clark equation) as special cases (see detailed discussion later).

**Figure 1:**
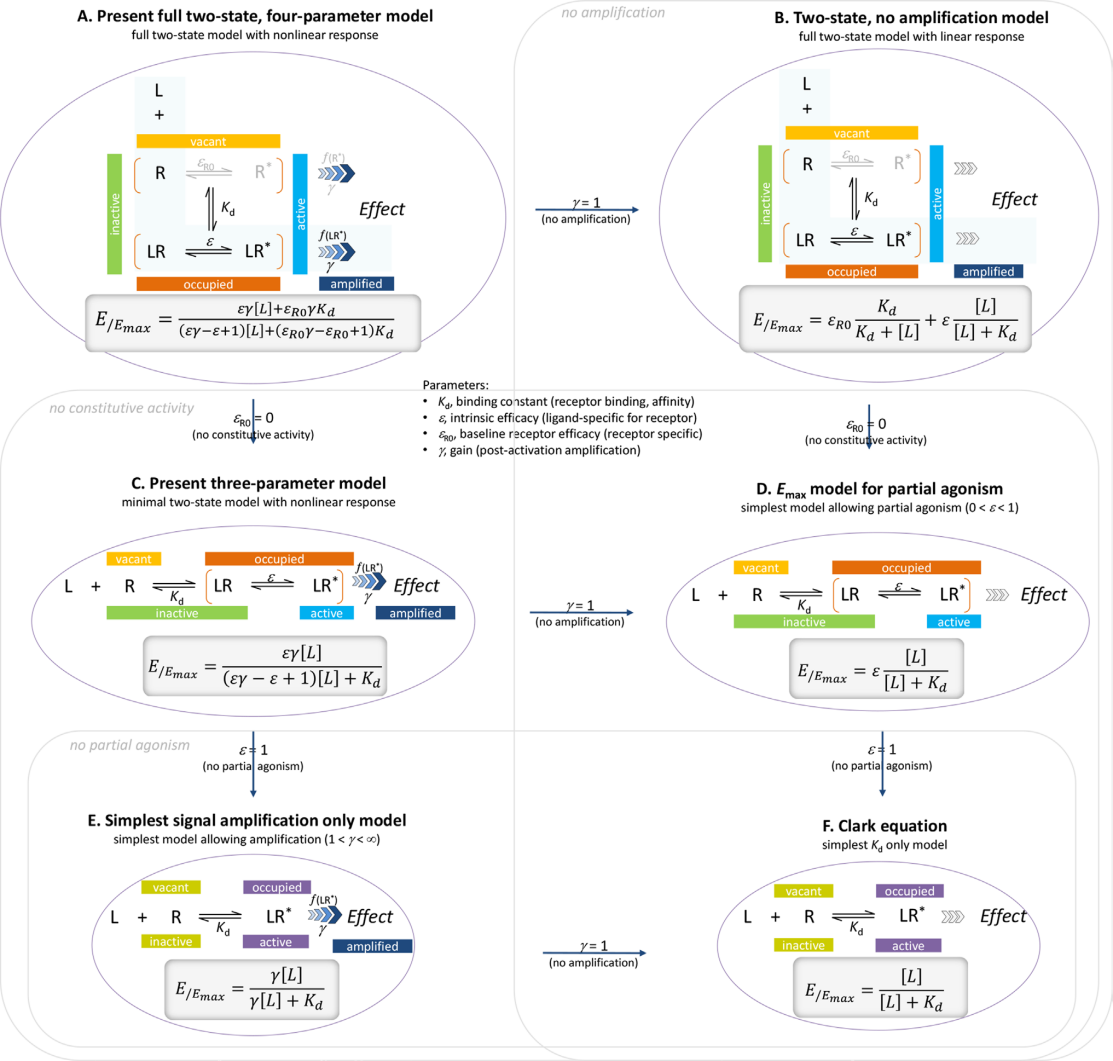
The present general two-state SABRE receptor model **(A)** and its consecutively nested simplifications down to the Clark equation **(B **to **F)**. For each model, a schematic illustration of its basic assumption on ligand binding and receptor activation is shown together with the corresponding quantitative form relating the fractional effect (*E*/*E*_max_) to the ligand concentration [L].

Here, a general two-state model of receptor function is proposed that incorporates constitutive activity into our previous model (Buchwald, 2017). It integrates three distinct processes, each characterized with its own parameter: 1) receptor binding, characterized by *K*_d_, the equilibrium dissociation constant used for binding affinity; 2) receptor activation, characterized by an (intrinsic) efficacy parameter *ε* (plus a baseline receptor efficacy *ε*_R0_ for receptors with constitutive activity); and 3) post-activation signal transduction (amplification), characterized by a gain parameter *γ*. The final form can fit complex fractional response versus occupancy data, but it is also a true generalized model that can collapse back to consecutively nested simpler ones for special cases of its parameters (**Figure 1**).

## Present Model: Two-State Model With Binding, Efficacy, and Amplification Parameters

### Main Concepts and Equations

In agreement with the generally accepted picture, the present model assumes that to produce a response, a ligand must first bind to a receptor and then activate it to initiate some downstream signaling. The main assumption of the two-state receptor theory that ligand-bound (occupied) and active receptor states do not fully correspond is maintained, but a slightly different parametrization is employed to achieve more intuitive affinity (binding) and efficacy (activation) quantification. Binding of the ligand is assumed to alter the likelihood of activation; i.e., an induced fit type model is presumed. Receptors can be either active or inactive in their ligand-free or ligand-bound forms; however, the corresponding probabilities (i.e., times spent in the corresponding conformations) can be quite different. Ligand-free and ligand-bound states will be considered as an equilibrium ensemble of active and inactive conformations present. In general, a ligand-free receptor is overwhelmingly in an inactive conformation, and in cases where there is no constitutive activity, it is entirely so. Binding of an agonist, which is governed by its affinity parameter *K*_d_, shifts the equilibrium toward the active state. The ability of a bound ligand to do so is characterized by an (intrinsic) efficacy parameter, *ε*. For receptors with constitutive activity, a basal receptor efficacy, *ε*_R0_, is used to account for baseline activation in absence of a ligand. The signal (effect) generated by the active receptor can be amplified downstream, and this is characterized by a pathway-specific gain parameter *γ*. Hence, the most general form of the model uses four parameters: *K*_d_, the equilibrium dissociation constant characterizing binding affinity; *ε*, an (intrinsic) efficacy characterizing the ability of bound ligand to activate the receptor (0≤*ε*≤1); *ε*_R0_, a basal receptor efficacy characterizing constitutive activity (if present); and *γ*, a gain (amplification) parameter characterizing the nonlinearity of (post-activation) signal transduction (1≤*γ*<∞). As explicit incorporation of a signal amplification parameter is a main novelty, one can designate this model as Signal Amplification, Binding affinity, and Receptor activation Efficacy (SABRE). The overall schematic of the present model in its most general form and the corresponding quantitative equation are shown below. The same, together with all its successive nested simplifications corresponding to special cases are shown in **Figure 1**.

(1)
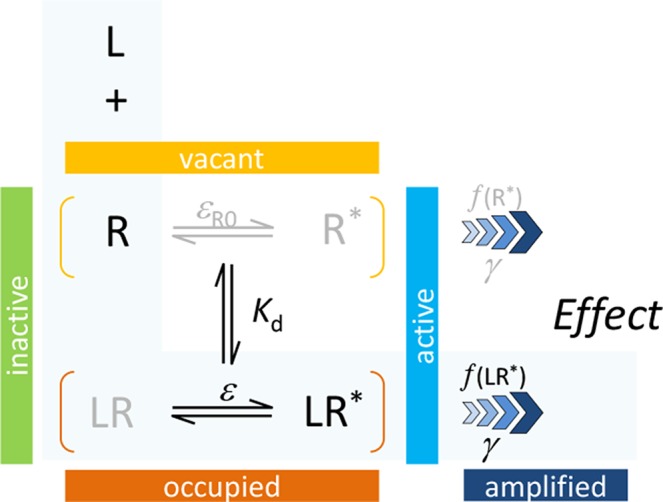


(2) $$E_{/E_{max}}=\frac{\varepsilon \gamma [L]+\varepsilon_{R0}\gamma K_{d}}{(\varepsilon \gamma -\varepsilon +1)[L]+(\varepsilon_{R0}\gamma -\varepsilon_{R0}+1)K_{d}}$$

For cases with no constitutive activity (*ε*_R0_ = 0, no active unbound receptor, R*), this reduces to the three-parameter model previously introduced corresponding to the portion highlighted in light blue above and represented by the bottom two rows of **Figure 1** (models C–F) (Buchwald, 2017),

(3)
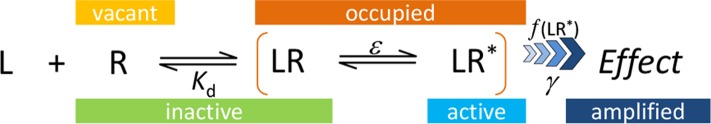


(4) $$E_{/E_{max}}=\frac{\varepsilon \gamma [L]}{(\varepsilon \gamma -\varepsilon +1)[L]+K_{d}}$$

Details of model parametrization and assumptions are discussed below, followed by highlights of its advantages and illustrative applications for complex response versus occupancy data. A detailed derivation of Equation 2 is included in **Appendix 1**.

### Parametrization

#### Binding Parametrization, *K*_d_

To produce a response, a ligand must first bind to the receptor. Binding parametrization is typically achieved via the widely used equilibrium dissociation constant (*K_d_*), which is measured in units of concentration (usually molarity, M). In the simplest one-state model, where all occupied receptors are active and that forms the basis of the widely used one-parameter Clark equation (**Figure 1F**), *K*_d_ is expressed as a function of the equilibrium concentrations of the species involved as:

(5) $$K_{d}=\frac{\left[L][R\right]}{\left[LR^{*}\right]}$$

It is important to remember that *K* is not just an arbitrary model parameter: it is an entity measurable in equilibrium or kinetic binding assays, and it is related to the Gibbs free energy of binding (*ΔG*) via the well-known thermodynamic equation:

(6) $$K=e^{-\frac{\Delta G^{0}}{RT}}$$

Here, *T* is the absolute temperature and *R* the universal gas constant, *R* = *k*_B_ *N*_A_ = 8.314 J/K·mol. Accordingly, to bind with 1 nM affinity (*K*_d_ = 10^–9^ M) at physiological temperature (*T* = 310 K), a ligand requires a free energy of binding of *ΔG* ^0^ = –*RT*·ln*K*_d_ = 53.4 kJ/mol (12.8 kcal/mol).

**Figure 2:**
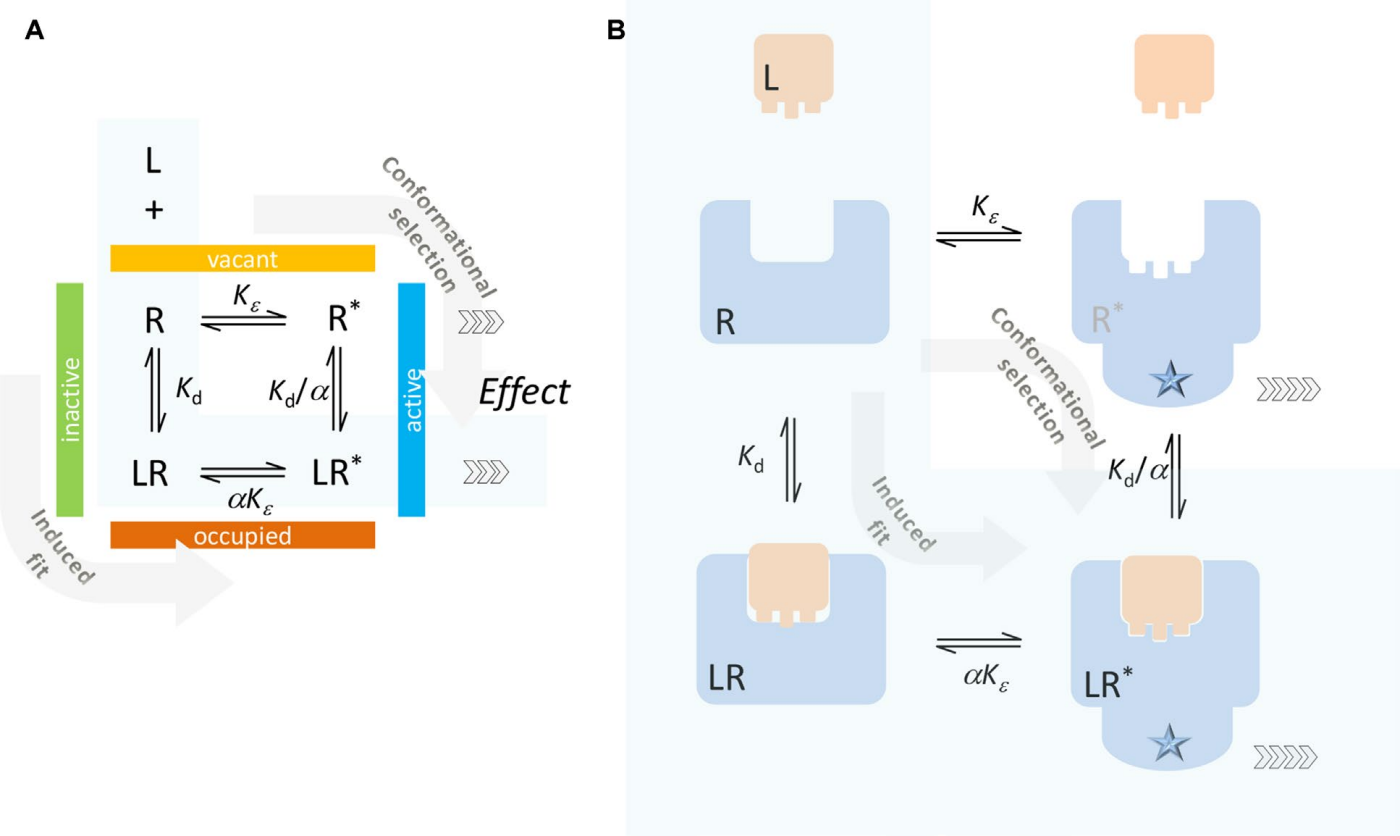
Typical two-state receptor model with assumption of different binding affinities for the inactive and active forms of the receptor (R and R*, respectively). Schematics **(A)** and a simplified illustration of the corresponding processes **(B)** are shown. The ligand can bind to the inactive form of the receptor (R) and contribute to its activation (induced fit, conformational induction) or bind to the active form (R*) and lock it preferentially in that conformation (conformational selection). The case corresponding to the minimal two-state theory (no constitutive activity, i.e., ligand-free receptor has no active form) is highlighted with a light blue background.

The present model uses a similar definition for *K*_d_. It differentiates between active and inactive states (denoted by an asterisk; R vs. R* and LR vs LR*), but contrary to most two-state models that distinguish between binding affinities for the active and inactive states (e.g., *K*_d_ and *K*_d_/*α*; **Figure 2**), it considers ligand-bound and ligand-free states as ensembles of conformations characterizable by the same *K*_d_. Hence, *K*_d_ here represents an average binding constant for the ensemble of active and inactive receptor forms that the ligand effectively sees. With this assumption, the definition of *K*_d_ will rely on the total concentration of occupied and unoccupied receptors, which in the case of the full two-state model (**Figure 1A**) becomes

(7) $$K_{d}=\frac{\left[L]([R]+[R^{*}]\right)}{\left([LR]+[LR^{*}]\right)}$$

This is a macroscopic equilibrium constant measurable in equilibrium binding assays that assess total binding to the receptor. In case of the minimal two-state model, where there is no constitutive activity (**Figure 1C**, [R*] = 0), it reduces to the previously used form (Buchwald, 2017):

(8) $$K_{d}=\frac{\left[L][R\right]}{\left([LR]+[LR^{*}]\right)}$$

**Figure 3:**
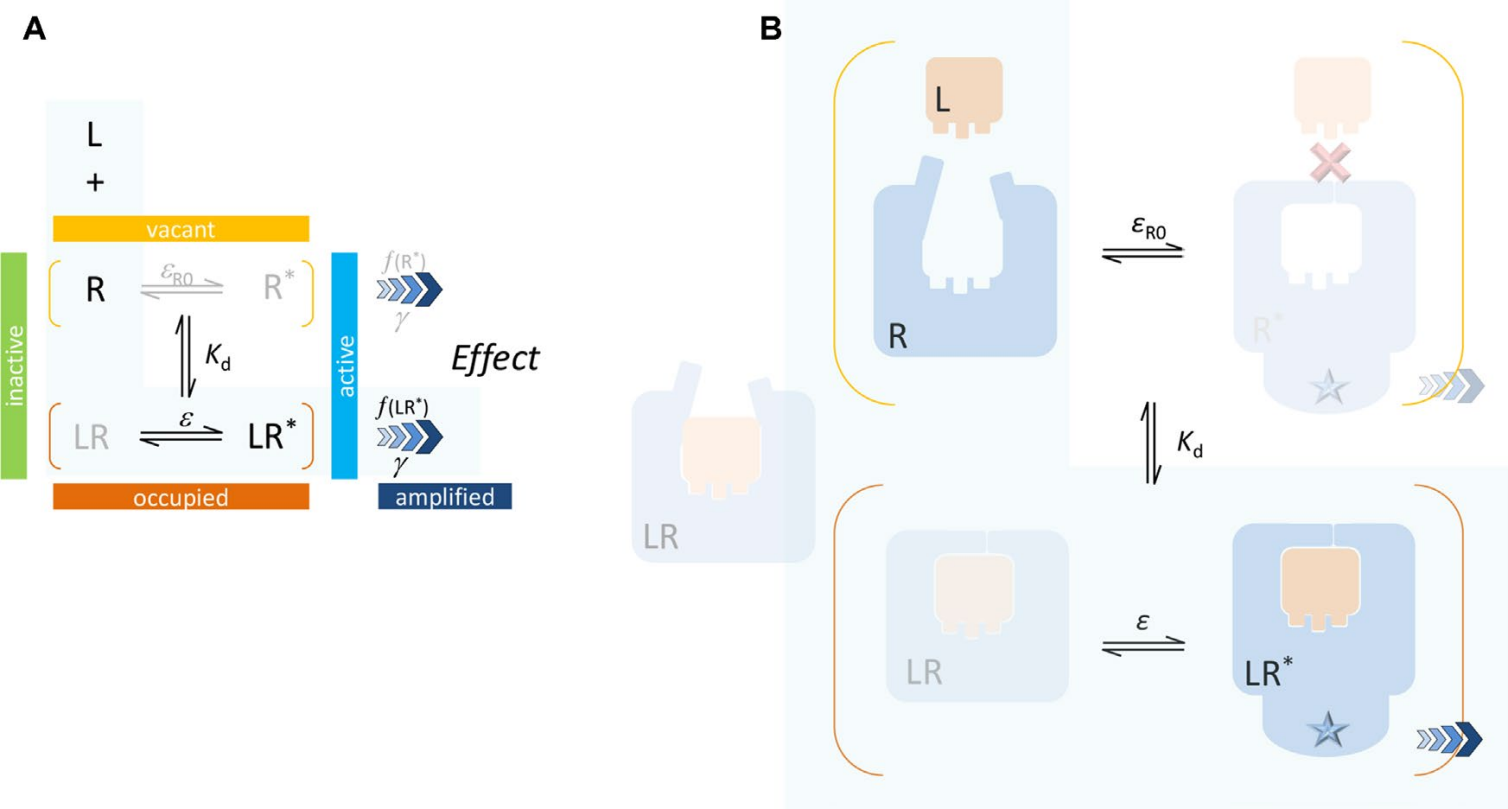
Present two-state model: schematics **(A)** and a possible simplified illustration **(B)**. A single binding affinity (*K*_d_) is assumed that represents an ensemble average of the binding to the active and inactive conformations. In most cases, direct binding of small-molecule ligand to the active R* form is unlikely (as indicated by the red X mark) since the ligand-binding domain (LBD) sites are buried deep inside the receptor and are not directly accessible from the surrounding environment. Again, the case corresponding to the minimal two-state theory (no constitutive activity, i.e., ligand-free receptor has no active form) is highlighted with a light blue background.

Binding, characterized by *K*_d_, is not just snapping of a ligand into a rigid site (“key-in-the-lock”), which corresponds to that of the active conformation (inactive for an antagonist), but it also involves change in energy and possibly adaptation of the site to accommodate the ligand (“hand-in-the-glove”). The corresponding conformational shift (toward active states) leads to change in the downstream activation (induced fit) (**Figure 3**). A key advantage of this definition is that *K*_d_ represents ensemble averages for the bound and unbound receptor forms and becomes independent from the value of the efficacy parameter (Buchwald, 2017). Another one is that it avoids the loop-related thermodynamic constrains (i.e., need for a same *α* for both *K*_d_ and *K_ε_* in **Figure 2**) as will be discussed in the section Mechanism of Receptor Binding and Activation.

#### Efficacy Parametrization, **ε**

The need for efficacy parametrization, besides that for binding, arose from the recognition of partial agonism, i.e., the existence of compounds that can occupy all receptors without achieving full activation. Well-known partial agonist examples include prenalterol (versus the full agonist adrenaline at β-adrenoceptors), pilocarpine (versus acetylcholine at muscarinic receptors), and impromidine (versus histamine at H_2_ receptors) (see **Supplementary Figure S1** for representative chemical structures). Here, efficacy parametrization is done with an *ε* parameter that represents the fraction of ligand-bound receptors that are active (Buchwald, 2017):

(9) $$\varepsilon =\frac{\left[LR^{*}\right]}{\left[LR]+[LR^{*}\right]}$$

**Figure 4:**
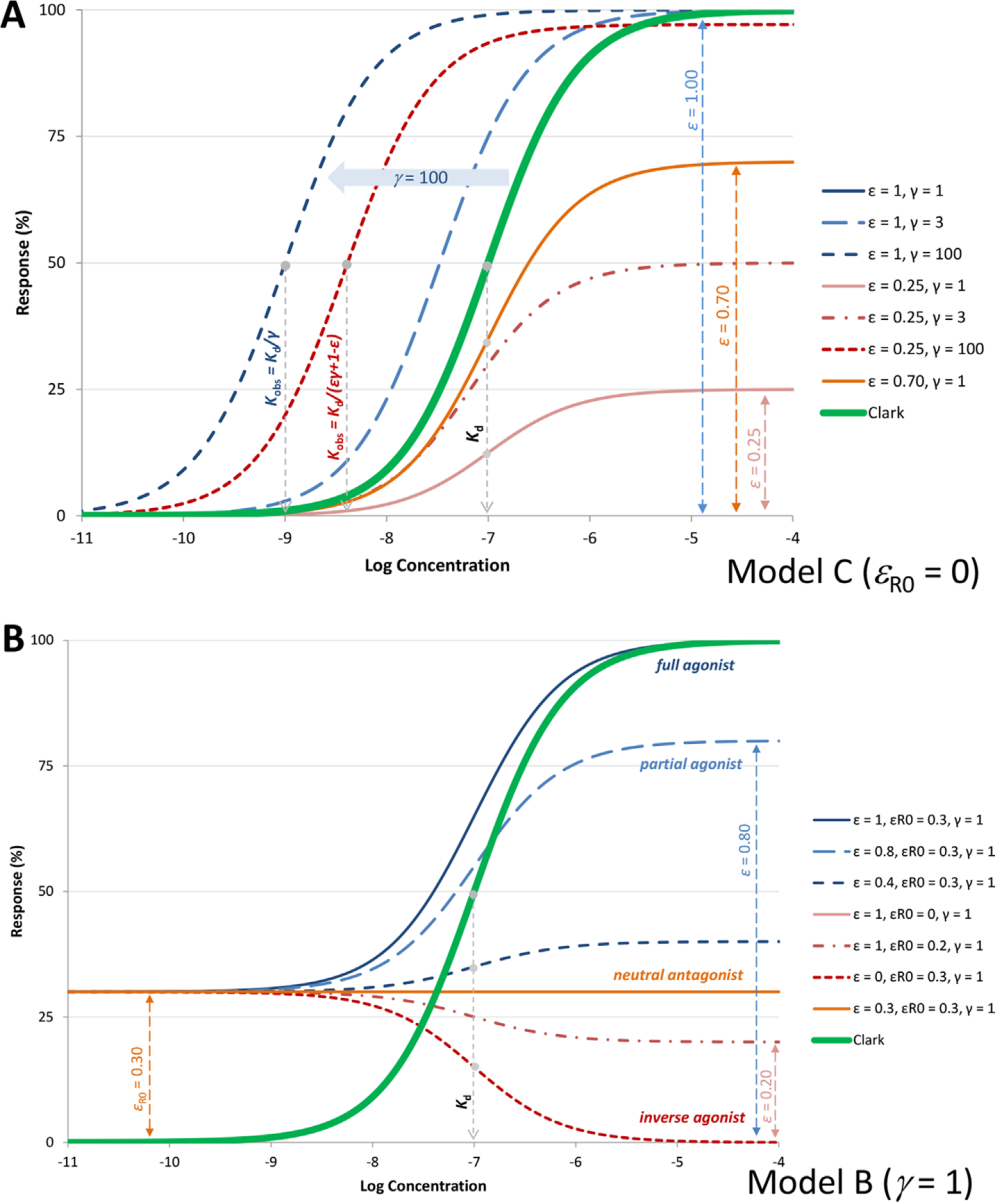
**(A)** Semilog concentration–response curves with the present model for a receptor without constitutive activity (Equation 4, **Figure 1C**) and ligands with 100 nM affinity (*K*_d_ = 10^–7^ M). Response curves shown are for a full and a weak partial agonist (*ε*=1, blue lines and *ε*=0.25, red lines, respectively) at different amplifications (*γ*=1, 3, and 100; denoted with full and dashed lines, respectively). Another partial agonist without amplification is also included (*ε*=0.70, *γ*=1 orange line) for comparison. Note that with the present model, the basic parametrization (*ε*=1, *γ*=1) fully reproduces the Clark model (blue and double green lines completely overlap), which could not be done with previous models such as the operational model. **(B)** Illustrative response curves with the present model for a case with constitutive activity (*ε*_R0_=0.3) and no amplification (**Figure 1B**). Response curves for full, partial, and inverse agonists as well as a neutral antagonist with the same affinity as in A (*K*_d_ = 10^–7^ M) are shown as obtained with the efficacy parametrization of the present model.

Hence, efficacy *ε* as defined here is a unitless parameter (but not an equilibrium constant such as *K_ε_*) and is an intrinsic efficacy in the sense that it is a characteristic of the ligand L (for a given receptor R). It ranges from 0 for an antagonist that can occupy all receptors but produces no effect to unity (1) for a full agonist that converts all occupied receptors to active ones. The activity here is the one measured immediately post-receptor; downstream signal amplification can complicate things as response from a partial agonist can be amplified to a maximum final response (see discussion later). A set of representative response curves obtained for a fixed binding affinity (*K*_d_) but different efficacies *ε* are shown in **Figure 4A**.

Note that the efficacy *ε* as defined here is different from the efficacy *e* as defined by Stephenson, which was introduced to measure the ability of a drug to produce a response in a tissue. In Stephenson’s definition, the stimulus was *S* = *e*[LR*]/[R_tot_], and *e* could have values from 0 up to infinity (Stephenson, 1956). In fact, *ε* is more similar to the efficacy as defined by Furchgott (*ϵ*_F_ = *e*/[R_tot_]) representing “the capacity of a ligand to initiate stimulus from one receptor” (here, as measured right after the receptor before additional signaling steps). It is also similar to the intrinsic activity *α*_A_ introduced by Ariëns (1954), which is the ratio of the maximum response produced by the partial agonist to that produced by the full agonist and is ultimately quantifying “effect per unit of pharmacon–receptor complex” (as long as there is no amplification).

With these assumptions, the fractional response (effect) *f*_resp_, which under the assumptions of no amplification (**Figure 1D**) equals the fraction of activated receptors *f*_act_, can be expressed as a function of ligand concentration only as (Buchwald, 2017):

(10) $$f_{resp}=E_{/E_{max}}=\frac{\left[LR^{*}\right]}{\left[R_{tot}\right]}=\frac{\varepsilon \frac{\left[R][L\right]}{K_{d}}}{[R]\left(1+\frac{\left[L\right]}{K_{d}}\right)}=\varepsilon \frac{\left[L\right]}{[L]+K_{d}}$$

This is essentially equivalent with a Clark equation that includes a scalable maximum (*E*_max_ model for partial agonism; **Figure 1D**). It is also equivalent with the model proposed by Ariëns (e.g., Equation 12 in Ruffolo, 1982) incorporating an intrinsic activity term (*α*_A_). Equation 10 links the definition of the maximum achievable effect (response) for a partial agonist, *f*_resp,max_ = *ε*, to a two-state model (**Figure 1D**). With this definition, *ε* corresponds to the fraction of maximum activation a partial agonist can achieve as compared to the full agonist—as measured right after the receptor (to avoid possible confounding effects created by downstream amplification; see later). For full agonists, *ε* = 1 and the above equation correspond directly to the Clark equation for response (Clark, 1926; Clark, 1933) (**Figure 1F**), which is mathematically equivalent with the Hill–Langmuir equation for ligand binding (Hill, 1909) and a special case of the versatile Hill equation (Hill, 1910) often used in pharmacological and other applications (Goutelle et al., 2008; Gesztelyi et al., 2012).

#### Incorporation of Constitutive Activity

Since the introduction of the concept in the late 1980s (Costa and Herz, 1989), it is now well recognized that certain G-protein–coupled receptors (GPCRs) can be active even in the absence of an agonist (have constitutive signaling activity) and that some ligands can act as inverse agonists (i.e., reduce the activity of the ligand-free receptor) (Bond and Ijzerman, 2006). To incorporate such constitutively active receptors into the formalism of the present model (**Figure 1A**), a baseline receptor efficacy (*ε*_R0_) is introduced along lines similar to the definition of *ε* (Equation 9), i.e., the fraction of unbound receptors that are active:

(11) $$\varepsilon_{R_{0}}=\frac{\left[R^{*}\right]}{\left[R]+[R^{*}\right]}$$

However, contrary to *ε*, which is a ligand characteristic, *ε*_R0_ is a receptor and not a ligand characteristic. For such receptors, ligands known as inverse agonists reduce the signaling output below that of the basal state. In the present formalism, they will have efficacies that are smaller than this baseline receptor efficacy, *ε*_inv.agon._<*ε*_R0_. Partial agonists remain those compounds that generate a response, albeit a submaximal one even at concentrations that saturate all receptor sites (*ε*_R0_<*ε*<1), and so-called neutral antagonists do not discriminate between the two conformers (*ε*_R0_=*ε*) and thus can block agonist, partial agonist, and inverse agonist activities. Some representative response curves that can be obtained with this parametrization are shown in **Figure 4B**, and further discussion is included later.

#### Gain (Signal Amplification) Parametrization, γ

Some kind of signal amplification is needed to account for the existence of “spare receptors” or “receptor reserves”: cases where almost maximal response can be achieved by occupying only a (small) fraction of all receptors. The concept has been introduced in the mid-1950s (Nickerson, 1956; Stephenson, 1956) with a method for its quantification described by Furchgott about a decade later (Furchgott, 1966; Furchgott and Bursztyn, 1967). Well-known extreme cases include, for example, the response of the human calcitonin receptor type 2 to calcitonin (where 20% occupancy already produces close to 100% response) (Chen et al., 1997) and guinea pig ileal response to histamine (where 2% occupancy already produces close to 100% response) (Kenakin and Cook, 1976; Adham et al., 1993; Kenakin, 2018b). Another example is the stimulation of β-adrenergic receptors in the heart by epinephrine where half-maximal increase of muscle contractility already occurs at 1% to 3% receptor occupancy in rats and at 10% to 20% occupancy in humans, and the effects saturate well before the receptors do (Brown et al., 1992). Detailed quantifications, including for different ligand series, have been done for several cases, typically GPCRs, such as the muscarinic acetylcholine (Furchgott and Bursztyn, 1967; Harden et al., 1986; Eglen and Whiting, 1987), opioid (Chavkin and Goldstein, 1984; Adams et al., 1990; Fox and Hentges, 2017), dopamine (Meller et al., 1987), 5-hydroxytryptamine (5-HT) (Meller et al., 1990), A_1_-adenosine (Dennis et al., 1992; Morey et al., 1998), and cannabinoid (Gifford et al., 1999) receptor systems.

Most commonly, such cases are due to strong signal amplification via a downstream cascade of saturable functions provided by second messengers or other systems (Strickland and Loeb, 1981; Koshland et al., 1982; Ferrell, 1996). Activation of adenylate cyclase and production of cyclic AMP (cAMP) as second messenger followed by further downstream cascading is a well-known example. For example, the mechanism by which epinephrine (acting on a myocyte) or glucagon (acting on a hepatocyte) sets off a cascade of phosphorylation leading to the production of glucose involves downstream amplification of the initial signal by several, possibly up to eight orders of magnitude (Nelson and Cox, 2012). As a result, epinephrine concentrations as low as 10^–10^ M in the blood can stimulate liver glycogenolysis and release of physiologically adequate levels of glucose. Such a small epinephrine stimulus can generate intracellular cAMP concentrations of 10^–6^ M (already a ∼10,000-fold gain) followed by three more catalytic steps leading to release of glucose via another approximately 10,000-fold amplification (Lodish et al., 2003). In many ways, the resulting cascade mechanisms of signal amplification in biological systems resemble those used in electronic circuits (Grubelnik et al., 2009). Very strong amplification is not always desirable as amplification of noise-level signals has to be avoided (Bialek and Setayeshgar, 2005).

In the present model, pathway-specific signal amplification is incorporated explicitly using a separate gain parameter *γ*. This is an important novel component that has not been explicitly included as such in previous models like the operational (Black and Leff) or the minimal two-state (del Castillo–Katz) model. Amplification is built in via use of a nonlinear hyperbolic-type response function (Buchwald, 2017). Hyperbolic relationships between fractional occupancy and fractional response have been found in several cases (see examples later), justifying the use of such functions. Use of hyperbolic functions also has the advantage that even if there is a cascade of such sequential saturable signal amplification functions with the output of one serving as input for the next one (as is often the case in biological systems), the overall response can be represented by a single hyperbolic-type response function (Trzeciakowski, 1999a). The nonlinear response (amplification) function used here is a classic hyperbolic function, just as it is in the operational model. However, to be able to reach its asymptotic limit, not [LR*] is used as its input, because [LR*] cannot be higher than [R_tot_] and, hence, cannot cover the asymptotic part of the response function that is beyond the [R_tot_] value, but its odds-ratio type (De Muth, 2014) transform: *Λ* = *p*/(1–*p*) (see Buchwald, 2017 for further details). In other words, the input for the hyperbolic function here is not [LR*], but *Λ* = [LR*]/([R_tot_] − [LR*]) = *f*_act_/(1 − *f*_act_), with *f*_act_ = [LR*]/[R_tot_] representing the fraction of active receptors. For full agonists, *f*_act_ corresponds to the fraction occupied *f*_occup_; *f*_act_ = *f*_occup_ if *ε* = 1. This extends the range of the input from 0–1, which is the range for *f*_act_ (i.e., [LR*]/[R_tot_]), to 0–∞.^1^ Hence, the response function linking *E* to [LR*] will be:

(12) $$E_{/E_{max}}=F([LR^{*}])=\frac{\Lambda }{\Lambda +\frac{K_{\gamma }}{\left[R_{tot}\right]}};\Lambda =\frac{\left[LR^{*}\right]}{\left[R_{tot}]-[LR^{*}\right]}=\frac{f_{act}}{1-f_{act}}$$

After some transformations and introduction of *γ*=[R_tot_]/*K_γ_*, this leads to the final general form for the present three-parameter model (**Figure 1C**) (Buchwald, 2017):

(13) $$E_{/E_{max}}=\frac{\varepsilon \gamma [L]}{(\varepsilon \gamma -\varepsilon +1)[L]+K_{d}}$$

Here, the just introduced *γ* parameter represent a unitless amplification (gain) factor. Since it is a gain, for all practical purposes, it has a value larger than unity, *γ*≥1. A set of illustrative response curves for a fixed value of *K*_d_ and different values of *ε* and *γ* are shown in **Figure 4A**. For a given ligand acting at a specific receptor, affinity (*K*_d_) and efficacy (*ε*) should be the same (as long as receptor-binding and activation are not influenced by the environment, and signaling bias, which will be discussed later, is not considered), but transduction (signal amplification) could be pathway and tissue-dependent. Hence, *γ*, and, in fact, both of its components, R_tot_ and *K_γ_*, can be tissue-dependent.

A slightly rearranged form of this equation provides a better understanding of the interplay between its parameters:

(14) $$E_{/E_{max}}=\frac{\varepsilon \gamma }{\left(\varepsilon \gamma -\varepsilon +1\right)}\frac{\left[L\right]}{[L]+\frac{K_{d}}{\left(\varepsilon \gamma +1-\varepsilon \right)}}$$

From here, it is clear that half-maximal activity (EC_50_) is observed at *K*_obs_ = *K*_d_/(*εγ*+1–*ε*), and maximum (fractional) effect achievable by a given ligand is *f*_resp,max_ = *εγ*/(*εγ*+1–*ε*). Hence, for a full agonist at the receptor (*ε *= 1), *K*_obs_ = *K*_d_/*γ* and *γ* is a straightforward multiplication factor causing a left-shift of the sigmoid response function by *γ* units on a semi-log scale. Thus, for such an agonist, signal amplification causes no change in the shape of the response on semi-log scale, just a left-shift by *γ* increasing the apparent potency *γ*-fold (as illustrated by the blue lines in **Figure 4A**). For an agonist that produces only partial activation at the receptor (*ε*<1), downstream amplification can increase the maximum response, and sufficiently strong amplification can transform such a partial agonist into an apparent full or close to full agonist. With a large enough *γ*, the maximum response, *f*_resp,max_ = *εγ*/(*εγ* + 1 − *ε*), can approach unity even for small *ε*s. However, for such partial agonists, the left shifts, (*εγ* + 1 − *ε*), are smaller than for a full agonist, (1·*γ* + 1 − 1 = *γ*), so that the change in the apparent EC_50_ (*K*_obs_) is less (red vs. blue lines in **Figure 4A**).

Finally, to include the effect of amplification for cases with constitutive activity (completely general case represented by model A in **Figure 1**), we will calculate the fraction of activated receptors *f*_act_, which is proportional with the effect right after the receptor, and use it as input for the amplification function. With the definitions of *ε*_R0_, *ε*, and *K*_d_, the receptor concentrations can be eliminated, and *f*_act_ can be expressed as a function of [L] as:

(15) $$f_{act}=\frac{\left[R^{*}]+[LR^{*}\right]}{\left[R_{tot}\right]}=\varepsilon_{R0}\frac{K_{d}}{K_{d}+[L]}+\varepsilon \frac{\left[L\right]}{[L]+K_{d}}$$

As before, this will serve as input via *Λ*=*f*_act_/(1–*f*_act_) for the present amplification function (Equation 12), resulting in the final form of the full four-parameter SABRE model:

(16) $$E_{/E_{max}}=\frac{\varepsilon \gamma [L]+\varepsilon_{R0}\gamma K_{d}}{(\varepsilon \gamma -\varepsilon +1)[L]+(\varepsilon_{R0}\gamma -\varepsilon_{R0}+1)K_{d}}$$

It can be seen from here that basal response ([L] = 0) is *ε*_R0_ *γ*/(*ε*_R0_ *γ* + 1 − *ε*_R0_), whereas the maximum effect for a ligand L ([L]→∞) remains *f*_resp,max_ = *εγ*/(*εγ*+1–*ε*) (see **Supplementary Figure S2** for an illustration of the effects of different *ε*_R0_ and *γ* on the response calculated with this equation). Rearranging this in a manner like that done for Equation 14 but also separating the basal response leads to

(17) $$\begin{array}{c} E_{/E_{max}}=\frac{\gamma (\varepsilon -\varepsilon_{R0})}{\left(\varepsilon \gamma -\varepsilon +1)(\varepsilon_{R0}\gamma -\varepsilon_{R0}+1\right)}\frac{\left[L\right]}{[L]+\frac{\left(\varepsilon_{R0}\gamma -\varepsilon_{R0}+1\right)}{\left(\varepsilon \gamma -\varepsilon +1\right)}K_{d}}\\ +\frac{\varepsilon_{R0}\gamma }{\left(\varepsilon_{R0}\gamma -\varepsilon_{R0}+1\right)} \end{array}$$

Hence, the half-point of the transition is at 

(18) $$K_{obs}=\frac{\varepsilon_{R0}\gamma -\varepsilon_{R0}+1}{\varepsilon \gamma -\varepsilon +1}K_{d}$$

Consistent with the true generalized nature of the present model, if there is no constitutive activity, *ε*_R0_ can be set to 0, and this recovers the simplified forms of the model that have been introduced earlier (Buchwald, 2017) (models C–F, **Figure 1**). Conversely, if there is no amplification (**Figure 1B**), *γ* = 1 and the total effect becomes:

(19) $$E_{/E_{max}}=\varepsilon_{R0}\frac{K_{d}}{K_{d}+[L]}+\varepsilon \frac{\left[L\right]}{[L]+K_{d}}$$

As illustrated in **Figure 4B** for some representative parameter values, this can describe concentration-response functions for full (*ε* = 1), partial (*ε*>*ε*_R0_), and inverse agonists (*ε*<*ε*_R0_), as well as neutral antagonists (*ε*=*ε*_R0_).

The combination of all these effects can result in quite complex concentration-response curves that may make fitting with well-defined parameters difficult (see below). Nevertheless, the present four-parameter model can account for cases where the mixture of signal amplification and partial agonism causes complex responses, and several specific examples will be discussed (see also Buchwald, 2017 for the case of competitive partial agonism). It can also account for the observation of different responses created by the same agonists if observed along different pathways (biased agonism; e.g., **Figures 13** and **14**) or at different vantage points on the same downstream pathway involving multiple amplification steps (e.g., **Figure 10**).

#### Parametrization Considerations and Model Selection Criteria

Before closing this section on parametrization, a few general modeling related considerations have to be highlighted. The present model can fit complex data such as those illustrated in the section Fit of Complex Fractional Response Versus Fractional Occupancy Data; however, use of its fully parameterized version (Equation 2 or even Equation 4) only makes sense if sufficient data are available, and if occupancy (binding) and response (effect) can be assessed independently and under sufficiently similar conditions. Since adequate model fitting requires the availability of 5–10 (well distributed) data points for each adjustable parameter (Knofczynski and Mundfrom, 2008; Austin and Steyerberg, 2015; Kenakin, 2018b), reliable fitting of the full model can only be accomplished if a sufficiently large number of data points are available. Along these lines, it has to be mentioned that the present model uses one more parameter than the operational model, e.g., three (*K*_d_, *ε*, *γ*) vs. two (*K*_d_, *τ*) for the case of no constitutive activity or four (*K*_d_, *ε*, *γ*, *ε*_R0_) vs. three [e.g., *K*_d_, *ε*, *χ* (Slack and Hall, 2012)] for cases with constitutive activity. This is relevant because obtaining well-defined parameter values could require more data points, and rigorous model selection criteria advocate the use of the simplest model that can still provide adequate fit (George, 2000; Myung and Pitt, 2004; Buchwald, 2005; Buchwald, 2007). However, the need for one extra parameter is more than compensated for by, on one hand, the intuitive nature of the present parameters (due to separation of efficacy in receptor activation from gain in signal amplification), and, on the other, the ability to use simplified forms with reduced number of parameters. Contrary to the operational models, with the present one, simplified forms can be recovered for special cases of its parameters, and these can and should be used on their own when adequate or when there is not enough data to support full parametrization. Note that Hill type extensions that involve an additional *n*_H_ parameter to account for more or less abrupt response functions are not discussed here.

## Model Assumptions and Comparisons With Other Receptor Models

### Mechanism of Receptor Binding and Activation

Currently, pharmacological receptors are classified into four main classes (from fast to slow mode of action): 1) ligand-gated ion channels (ionotropic receptors; part of the ion channel protein targets that also include voltage-gated and other ion channels), 2) G-protein-coupled receptors (GPCRs; metabotropic receptors), 3) catalytic receptors (including receptor tyrosine kinases), and 4) nuclear hormone receptors (Rang et al., 2015; Alexander et al., 2017). There are now significant biochemical, biophysical, and structural data that suggest that receptors (in particular, GPCRs) exist in a dynamic equilibrium between their inactive and active states. They can be active to some degree even in a ligand-free state (Changeux and Edelstein, 2011), and binding of ligands shifts this equilibrium (Hunyady et al., 2003). In general, ligand-free receptors are overwhelmingly in their inactive conformations; those that have no constitutive activity are entirely so. Binding of an agonist shifts the equilibrium toward the active state; binding of an inverse agonist shifts it even more toward the inactive state (**Figure 4B**).

The full two-state model, which allows for constitutive activity, is traditionally envisioned along the lines illustrated in **Figure 2**, whereby the ligand can either bind to the inactive form of the receptor and contribute to its activation (induced fit, conformational induction) or bind to the active form of the receptor and lock it preferentially in that form (conformational selection). The corresponding equilibrium constants are shown in **Figure 2** following a commonly used notation (e.g., Fig. 1 in Trzeciakowski, 1999b, Fig. 1 in Roche et al., 2013, or Eq. 21 in Kenakin, 2017) that assumes the ligand as having an *α*-fold different affinity for the active versus the inactive form. Due to loop-related thermodynamic considerations, there are only three independent parameters: *K*_d_ = [L][R]/[LR] and *K*_d_/*α* = [L][LR*]/[LR*] are the equilibrium dissociation constants for the inactive and active receptor forms, respectively; and *K_ε_* = [R*]/[R] and *αK_ε_* = [LR*]/[LR] are the equilibrium constants for the activation of the ligand-free and ligand-bound receptors.

The present SABRE model is based on a similar, but somewhat different formalism as summarized in **Figure 3**. It assumes that binding alters the propensity of the receptor for activation, but it does not consider different binding affinities for the active and inactive receptor forms. *K*_d_ represents an ensemble average of the binding to the active and inactive conformations in each state, and it is defined by Equation 7. Efficacy *ε* represents the fraction of ligand-bound receptors that are active (Equation 9), and the similarly defined baseline receptor efficacy *ε*_R0_ characterizes the fraction of ligand-free receptor that is active (Equation 11). Finally, the signal generated at the receptor can be amplified following a transduction function characterized by the gain parameter *γ* (Equation 12).

**Figure 5:**
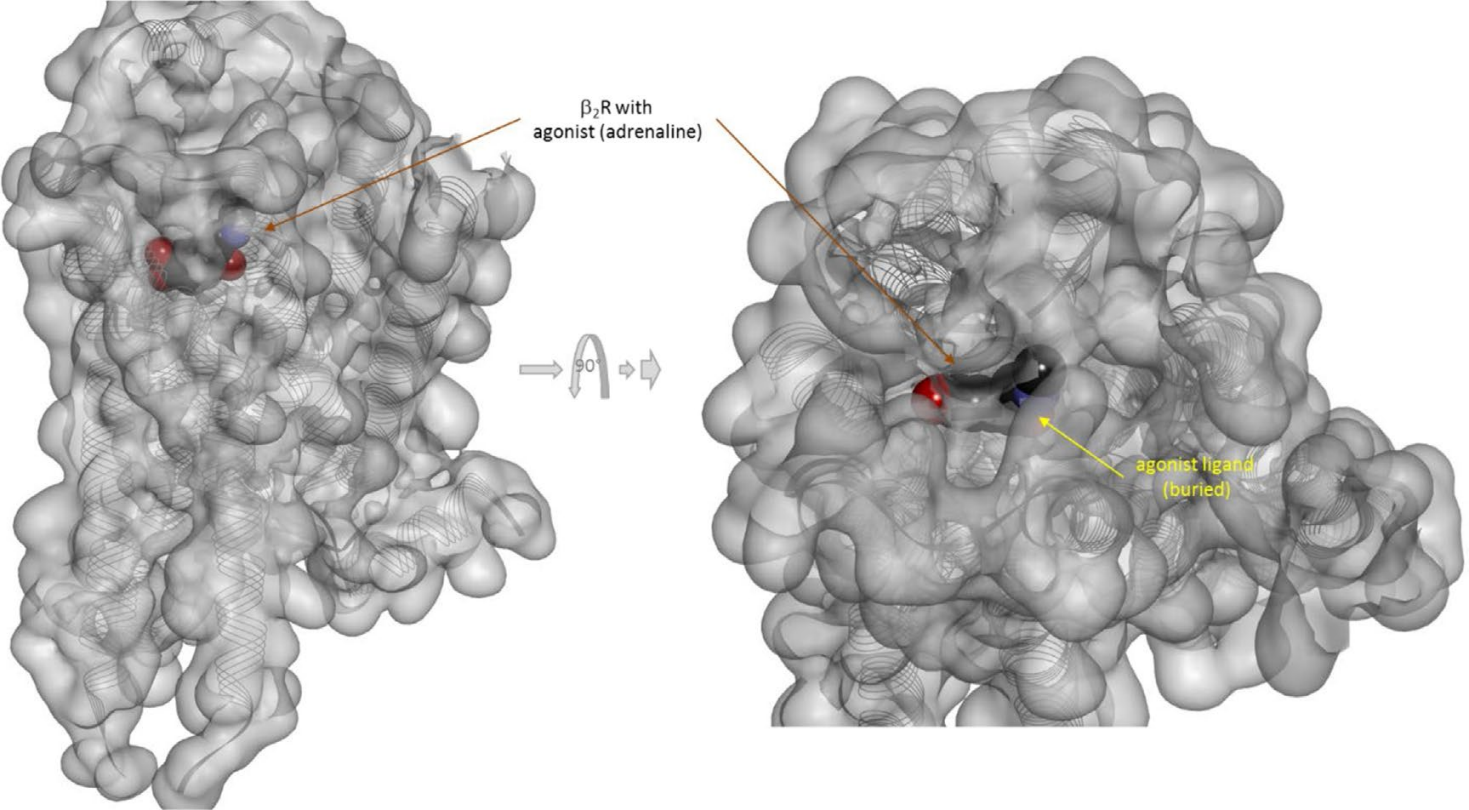
Three-dimensional structure of the agonist (adrenalin, epinephrine) bound active form of the β_2_-adrenergic receptor (a type Aα GPCR). The structure [PDB ID 4LDO (Ring et al., 2013)] is shown from two different perspectives (the one on the right being a 90° rotated and somewhat enlarged view from the top). The receptor is covered with a semi-transparent gray surface and the secondary protein structure indicated; the ligand is highlighted as a darker solid CPK structure. The ligand is somewhat faded as it is buried inside the receptor and obscured by the covering semitransparent surface; this is intended to illustrate that this position is not accessible for direct binding from outside.

**Figure 6:**
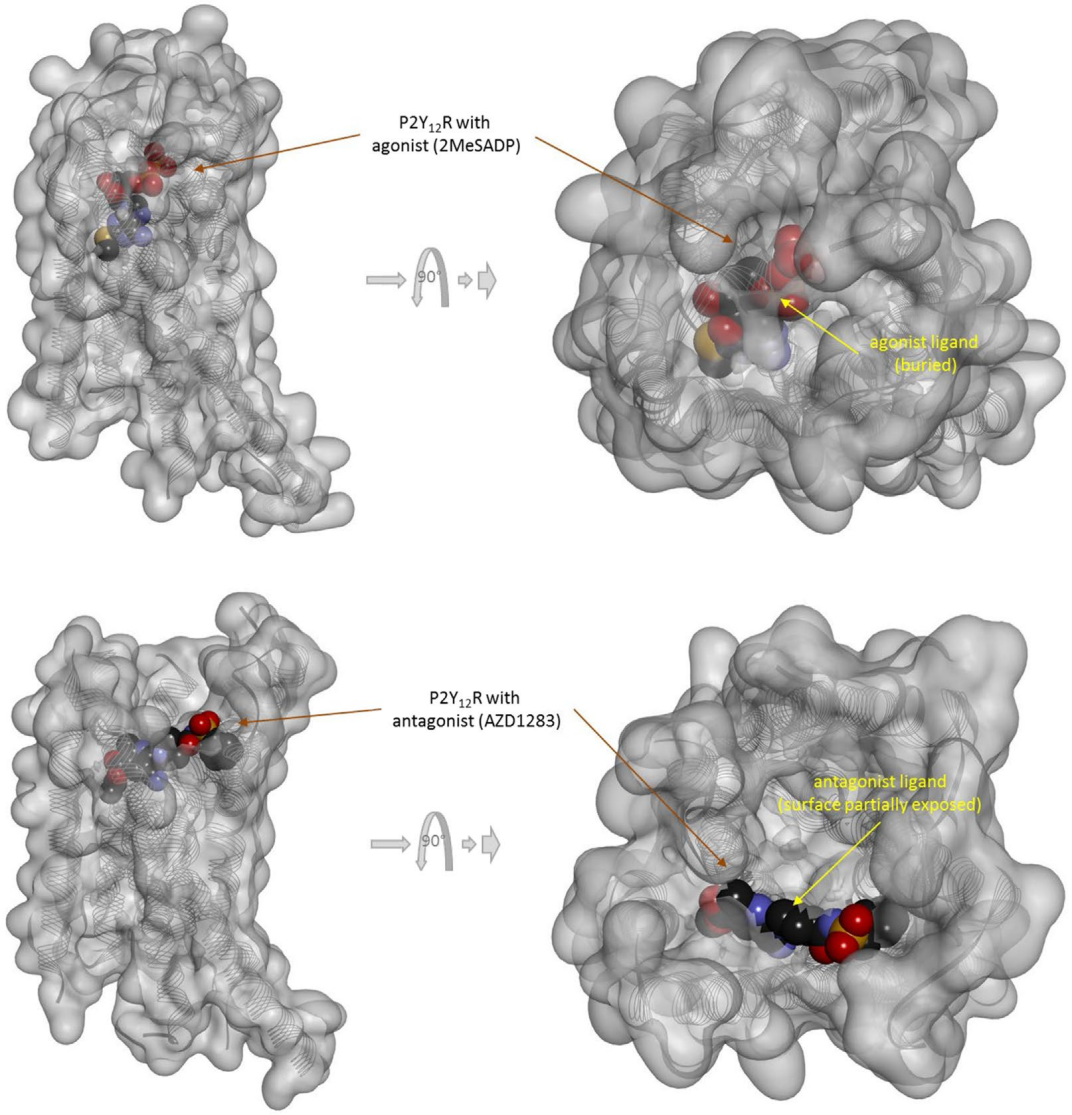
Three-dimensional structure of the agonist (2MeSADP; top) and antagonist (AZD1283; bottom) bound forms of the purinergic P2Y_12_ receptor (a type Aδ GPCR). Structures [PDB IDs 4PXZ and 4NTJ (Zhang et al., 2014a; Zhang et al., 2014b)] are shown covered with a semi-transparent gray surface and the secondary protein structure indicated; ligands are highlighted as darker solid CPK structures. Both are shown from two different perspectives with the one on the right being a 90° rotated and somewhat enlarged view from the top. The ligands are faded as they buried inside the receptor and are obscured by the covering surfaces; however, the antagonist (bottom) is less buried than the agonist (top) so that part of its surface is not covered and accessible from outside as indicated by its more vivid colors where directly visible.

**Figure 7:**
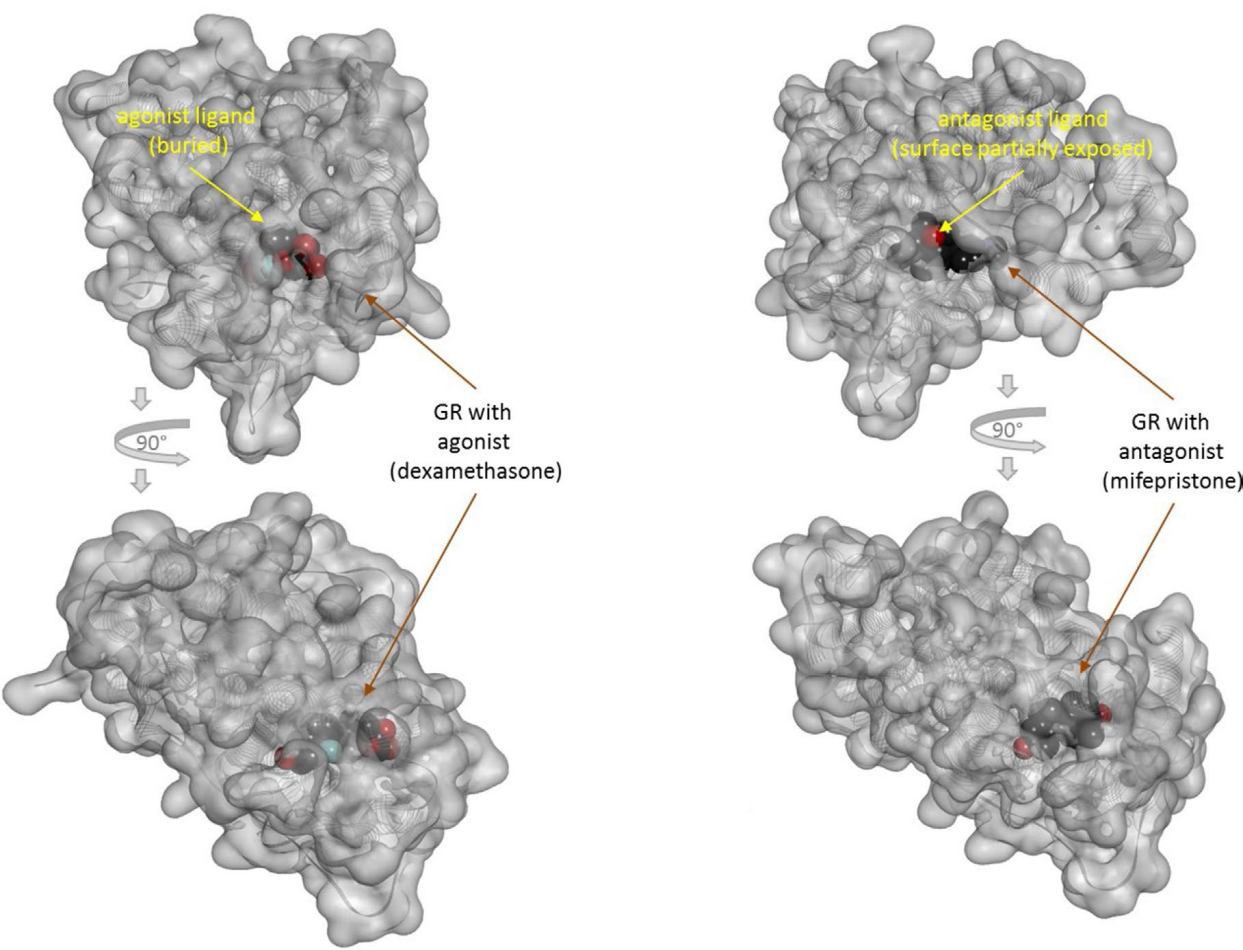
Three-dimensional structure of the agonist- (dexamethasone; left) and antagonist- (mifepristone; right) bound forms of the glucocorticoid receptor (a nuclear receptor). Structures [PDB IDs 1P93 and 1NHZ (Kauppi et al., 2003)] are shown from two different perspectives (the bottom one being a 90° rotated view as indicated by the arrows). Receptors are shown covered with a semi-transparent gray surface and the secondary protein structure indicated; the ligands are highlighted as darker solid CPK structures. The ligands are faded as they buried inside the receptors and are obscured by the covering surfaces; however, the antagonist (right) is less buried than the agonist (left) so that part of its surface is not covered and accessible from outside as indicated by its more vivid colors where directly visible.

**Figure 8:**
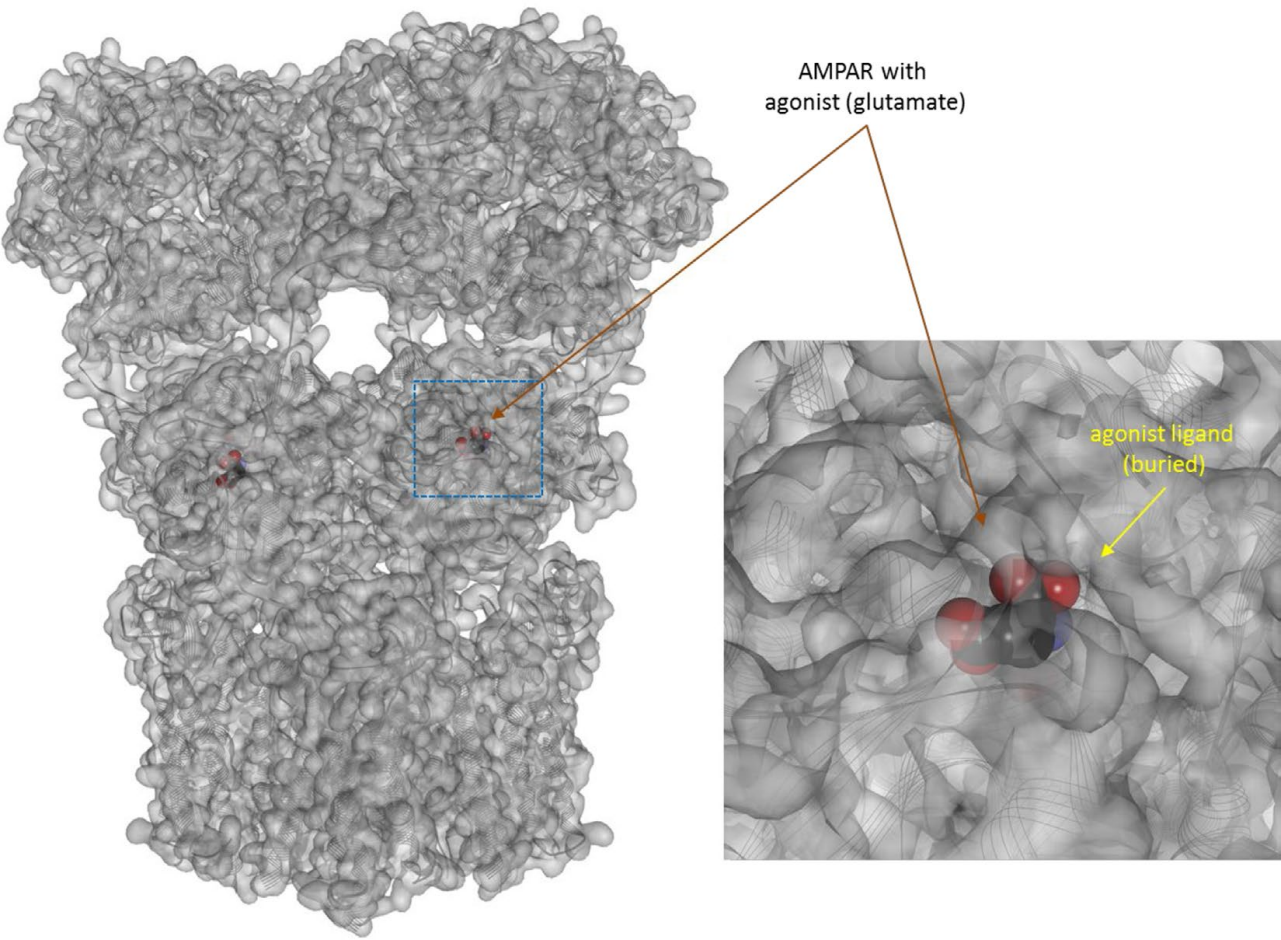
Three-dimensional structure of the agonist (glutamate) bound form of the AMPA receptor (a ligand-gate ion channel or ionotropic receptor). Structure [PDB ID 1P93 (Twomey et al., 2017)] is shown with the receptor shown covered with a semi-transparent gray surface and the secondary protein structure indicated; ligands are highlighted as darker solid CPK structures (inset shows a section around the bound ligand as an enlargement). As before, the ligands are faded as they buried inside the receptors and are obscured by the covering surfaces.

There is one other aspect of receptor binding that has to be considered. With a rapidly rising number of detailed receptor structures becoming available, including for several GPCRs, it is increasingly clear that most receptors in their active conformation have their small-molecule ligand-binding site buried deep inside. In many cases, binding sites, especially orthosteric ones, have been found to be fully buried (e.g., M2/3 muscarinic, β_2_ adrenergic, sphingosine-1-phosphate S1P, serotonin 5-HT, chemokine receptor CCR5, glutamate mGlu1, and others); in some, they are not and are partially solvent exposed (e.g., μ-opioid receptors and several peptide-activated class B GPCRs) (Manglik et al., 2012; Lee et al., 2015; Shonberg et al., 2015; Lu and Wu, 2016). A set of representative 3D examples is included for illustration: the agonist (adrenaline) bound active form of the β_2_-adrenergic receptor (a type Aα GPCR) (Ring et al., 2013) (**Figure 5**), the agonist (2MeSADP) and antagonist (AZD1283) bound forms of the P2Y_12_ receptor (a type Aδ GPCR) (Zhang et al., 2014a; Zhang et al., 2014b) (**Figure 6**), the agonist (dexamethasone) and antagonist (mifepristone) bound forms of the glucocorticoid receptor (a nuclear receptor) (Kauppi et al., 2003) (**Figure 7**), and the agonist (glutamate) bound form of the AMPA receptor (a ligand-gated ion channel or ionotropic receptor) (Twomey et al., 2017) (**Figure 8**). The need for fully buried binding sites is not coincidental, as they allow the bound ligand to interact with the receptor along its entire surface, so that relatively small volumes can focus multiple interactions (ionic, polar, hydrogen bond, and others) and achieve sufficiently strong binding with good enough ligand efficiency [binding energy per unit size (Hopkins et al., 2004)]. Such binding sites had to evolve for all endogenous small-molecule ligands to allow adequate potencies. Along these lines, it is also not coincidental that traditional drug targets, such as GPCRs, ion channels, or enzymes, are exactly those that have such well-defined cavities or clefts for binding their natural ligands as they can also be exploited for druggability purposes (Bodor and Buchwald, 2012; Zhu et al., 2012; Santos et al., 2017). Druggability requires sufficient potency (in general, sub-micromolar potency, e.g., EC_50_ < 1 μM) with existing small-molecule drugs having an average potency of 20 nM (Overington et al., 2006). This implies a need for strong enough binding energy (Δ*G* ^0^). The lack of well-defined binding pockets that make possible strong interactions along most of the ligand surface is a main reason why other therapeutic targets such as, for example, protein–protein interactions (PPIs) are so difficult to modulate by small molecules (Arkin and Wells, 2004; Scott et al., 2016; Bojadzic and Buchwald, 2018).

Because the ligand-binding domain (LBD) of most receptors in their active conformation is buried inside and is not accessible from the surrounding solvent, direct binding to the active form (conformational selection, selected fit) is an unlikely possibility for typical small-molecule ligands (highlighted by a red X in **Figure 3**). Since the LBD is not directly accessible from the outside when the receptor is in its active conformation (R*), it is unlikely that small-molecule agonists can simply “snap” in place and stabilize this conformation as required by a conformational selection mechanism (**Figure 2**). For the same reason, the assumption of a separate binding constant for the active state (e.g., *K*_d_/*α* in **Figure 2**) and the need for a thermodynamic loop seem unrealistic for most small-molecule bindings.

If, a tightly closed binding site is needed for efficient binding and activation, active conformations need to have a closed LBD that is not accessible directly from outside. The representative views showing fully buried agonists (**Figures 5**–**8**) were included here to highlight this. Further, this can also provide a plausible albeit simplified mechanism for the diminished ability of partial agonists to shift the equilibrium toward the active state (**Supplementary Figure S3**). Partial agonists or antagonists might hinder tight LBD closure (as illustrated in **Figure 6** or **Figure 7**) reducing their efficacy in activating the receptor. The “Venus flytrap domain” of class C GPCR provides a possible illustration as it fluctuates between open and closed conformations with agonists generally stabilizing the closed conformation and antagonists maintaining the open conformation (Geng et al., 2013; Koehl et al., 2019). In medicinal chemistry, it is well known that partial agonists and antagonists typically maintain certain important structural elements (pharmacophores) of the full agonists while also incorporating additional building blocks quite often resulting in larger molecular structures. Some well-known cases are illustrated in **Supplementary Figure S1**. Receptor binding is to a good degree size-dependent, and neither too small nor too large ligands can achieve the strongest binding (lowest energy) (Buchwald, 2008). The overall picture is certainly much more complex for various receptors, and this is a simplification of limited applicability. Nevertheless, it is mentioned as a possible simplified conceptualization that can be useful in some applications [“All models are wrong, but some are useful” (Box, 1979)]. Cases where full agonists induce LBD domain closure while partial agonists bind to a more open conformation have been shown, for example, for the AMPA receptor (**Figure 8**) (Jin et al., 2003; Twomey and Sobolevsky, 2018). On the other hand, structural information from co-crystallization studies with some GPCRs seem to suggest that their activation involves the translation of relatively modest structural changes within the ligand-binding site into larger-scale conformational shifts at the intracellular side of the receptor (Shonberg et al., 2014; Shonberg et al., 2015). Ultimately, these assumptions related to the activation and binding mechanisms (**Figure 3**, **Figure S3**) do not limit the general applicability of the formalism of the present model and its corresponding quantitative forms (**Figure 1**).

### Advantage Versus other Quantitative Receptor Models

The quantitative form of the present general SABRE receptor model (Equation 2 or Equation 4) has no striking beauty resulting from an elegant simplicity; nevertheless, it has several benefits compared to other complex quantitative models that are mostly based on the operational (Black and Leff) model (Black and Leff, 1983; Black et al., 1985):^2^

(20) $$E_{/E_{max}}^{op.mod.}=\frac{\tau [L]}{(\tau +1)[L]+K_{d}}$$

Most existing quantitative pharmacological models assume receptor functions along the lines of this operational model-based equation (Trzeciakowski, 1999a; Ehlert et al., 2011; Slack and Hall, 2012; Ehlert, 2015b; Copeland, 2016; Hall and Giraldo, 2018) with some additions needed for constitutive activity (Jenkinson, 2010; Kenakin, 2017; Kenakin, 2018b), including extension such as those by Ehlert and co-workers (Ehlert et al., 2011) or Hall and co-workers (Slack and Hall, 2012; Hall and Giraldo, 2018). However, there are noticeable disadvantages that hinder the widespread use of these *K*_d_ and *τ*-based equations, most of which are overcome by the present model. Although the overall forms of the present three-parameter model (Equation 4) and that of the operational model (Equation 20) are quite similar (with *εγ* here replacing *τ* of the operational model, plus an additional *ε* present in the denominator), the present parametrization provides several advantages over *τ*-based models. These will be briefly highlighted below.

#### Better Suited for Fitting by Nonlinear Regression

Transforming the classic form of the operational model (Equation 20) in a manner similar to that done for the present model (Equation 14 vs. 13) leads to 

(21) $$E_{/E_{max}}^{op.mod.}=\frac{\tau }{\tau +1}\frac{\left[L\right]}{[L]+\frac{K_{d}}{\tau +1}}$$

Hence, for the operational model, the maximum fractional response achievable by a ligand is *f*_resp,max_ = *τ*/(*τ*+1) and half-maximal activity occurs at EC_50_ = *K*_obs_ = *K*_d_/(1+*τ*). Because for full (or close to full) agonists, the maximum (fractional) response, *τ*/(*τ*+1), needs to be close to 1, *τ* needs to have large values, and those are difficult to obtain in a well-defined manner, as the *τ*/(*τ* + 1) fraction is no longer sensitive to changes in *τ* when approaching unity. Hence, fitting by nonlinear regression can result in large and ill-defined *τ* values, making the linked *K*_d_ values also badly defined. Since the calculated *K*_d_ is (*τ* + 1)-fold different from the observed *K*_obs_, the operational model (Equation 20) can end up not just with ill-defined, but also unrealistic *K*_d_ values that are essentially meaningless from a binding perspective. Hence, this model is difficult to fit for full or close to full agonists, and results can be cumbersome to interpret (see, e.g., Table 1 and Figure A1 in Buchwald, 2017 for specific illustrations). In agreement with this, mathematical identifiability analysis and simulation for the operational model has shown that when only functional assay data are available, only the transduction coefficient (*τ*/*K*_d_) and not *τ* can be estimated precisely (Zhu et al., 2018). There are indeed applications employing the operational model either only to determine unresolved *τ*/*K*_d_ ratios (Kenakin et al., 2012) or with experimental *K*_d_ values to constrain the regression (Rajagopal et al., 2011). Because in the present model, *ε* is restricted to the 0 to 1 range and *K*_d_ is independent of *ε*, fitting by nonlinear regression does not face these issues, and all parameters can be fitted in well-defined manner for partial and full agonists as long as there are sufficient data points. Several specific examples are included in the next chapter for illustration.

#### More Intuitive Parametrization

As highlighted by Equation 21, the *K*_d_ parameter of the operational model obtained from data fitting is different from the apparent (observed) *K*_obs_ as its value also depends on *τ*: *K*_obs_ = *K*_d_/(*τ*+1). Although this allows the concentration–response curve to shift from the concentration–binding curve, which is why such complex models are needed in the first place, it also makes *K*_d_ an empirical parameter not directly related to binding. As discussed above, for full or close to full agonists, *τ* needs to be large [e.g., *τ*>10 is needed for *E*_max_ = *τ*/(*τ*+1)>0.9], and *K*_obs_ values will be shifted considerably compared to *K*_d_—often to unrealistically high values that are clearly far from the actual binding affinity of the ligand. Hence, it has become accepted to use *K*_d_ as an empirical parameter not necessarily related to receptor binding, and in some implementations, such as in GraphPad Prism, full agonists are not fitted at all with this model. It has been pointed out that for the operational model, changes in binding (*K*_d_) and in conformation (*τ*) become indistinguishable for very efficacious agonists making interpretations difficult and cumbersome (Colquhoun, 1998).

Contrary to this, all parameters of the present model are straightforward, intuitive, and clearly related to their corresponding processes. Affinity (binding) is characterized by *K*_d_, the binding constant, and values measured in equilibrium assays can be used directly in the model. This *K*_d_ is uncoupled from the post-binding ability to activate the receptor (efficacy) as well as the strength of the post-activation amplification. Efficacy (ability to activate the receptor) is characterized by *ε*, a ligand-specific unitless parameter ranging from 0 (for a ligand that keeps all receptors inactive) to 1 (for an agonist that shifts all receptors into active state). Nonlinear transduction, due to post-receptor amplification (gain), is characterized by *γ*, a unitless parameter ranging from 1 (no amplification) to infinity.

#### Receptor Activation and Signal Amplification Are Separated

The present model also overcomes an essential hypothesis-related problem of the operational (Black and Leff) and del Castillo–Katz models, namely, that although the final equations are mathematically identical (Equation 20), they arrive at it from two conceptually different approaches that are both incomplete. On one hand, the minimal two-state (del Castillo–Katz) model (**Figure 2** without an R* state), which is now a generally accepted approach to describe switching of the receptor between active and inactive states, does not formally incorporate signal amplification (nonlinear transduction), which, as discussed, is known to exist. On the other, the operational (Black and Leff) model allows nonlinear response, but it is a single-state model that does not formally incorporate the possible existence of active and inactive ligand-bound receptor states (i.e., the possibility that not all occupied receptors are active), which is also known to exist. It makes up for this by limiting its output function to a maximum of *τ*/(*τ*+1). Consequently, these models, in fact, merge together two different effects in their *τ* parameter: the “intrinsic efficacy” of the (bound) ligand to activate the receptor, which can lead to partial activation (partial agonism) even with a linear response function, and the “efficacy” of the post-activation amplification downstream from the receptor, which can create fractional response in excess of the fractional occupancy (“receptor reserve”) and can be tissue- or organ-specific. These issues are overcome by the present model, by the introduction of separate efficacy *ε* and gain *γ* parameters that are also decoupled form the binding affinity, *K*_d_.

#### Reducibility to Previous and/or Simplified Models

Finally, another important advantage is that, contrary to previous models, the present one is a true generalized model: simplified forms can be recovered as special cases of its parameters, e.g., *ε*_R0_ = 0 for no constitutive activity, *γ* = 1 for no amplification (no receptor reserve), and *ε* = 1 for full agonism only (**Figure 1**). When adequate and/or when there is not enough data to support full parametrization, these simplified forms can and should be used on their own. The operational model, and consequently all of its extensions, cannot be reduced back to simpler forms, such as the *E*_max_ model for partial agonism or the Clark equation, as there are no *τ* values for which Equation 20 converts back to any of them (see Buchwald, 2017 for details). Therefore, one cannot transition back to simpler forms despite being in general desirable for complex models to be able to recover simpler ones for special cases of their parameters. In contrast, the present general model can be reduced back to a whole series of consecutively nested simpler forms (**Figure 1**). For example, *ε*_R0_ = 0 indicating no constitutive activity, reduces the general four-parameter model (**Figure 1A**) to the three-parameter model used before (**Figure 1C**). Further, if there is no post-receptor amplification, *γ* = 1, this model (**Figure 1C**) collapses back to an *E*_max_ model for partial agonists with efficiency *ε* (**Figure 1D**):

(22)
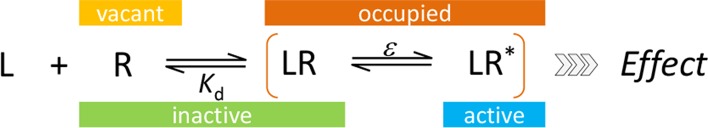


(23) $$E_{/E_{max}}=\frac{\varepsilon \cdot 1\cdot [L]}{(\varepsilon \cdot 1-\varepsilon +1)[L]+K_{d}}=\varepsilon \frac{\left[L\right]}{[L]+K_{d}}$$

Finally, if only full agonists are considered (*ε* = 1), all occupied receptors are active (**Figure 1F**), and the corresponding equation collapses back to the well-known Clark equation, which forms the basis of the entire quantitative receptor theory:

(24)
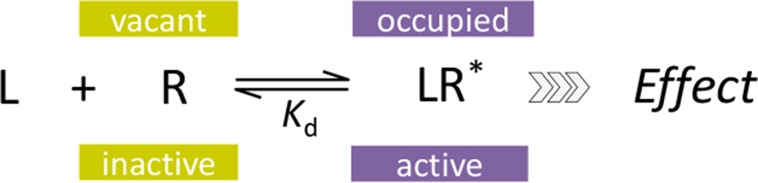


(25) $$E_{/E_{max}}=\frac{\left[L\right]}{[L]+K_{d}}$$

Consequently, one can start fitting with the simplest most adequate form corresponding to constrained parameters, and these constrains can be removed sequentially as needed based on the complexity of the data being fitted and the number of data points available. For the simplest case, one can start with *ε*_R0_ = 0 (no constitutive activity), *γ* = 1 (no amplification—fractional response overlaps with fractional occupancy), and *ε* = 1 (full agonism only) corresponding to a Clark model, and then remove these constrains as needed. On the other hand, the general model with all of its parameters released can fit complex cases where the fractional occupancy and the fractional response data are measured separately and do not correspond. In many cases, such data cannot be fit within the formalism of the operational model (e.g., to accommodate measured *K*_d_ values); a number of specific examples will be discussed below for illustration.

## Fit of Complex Fractional Response Versus Fractional Occupancy Data

In systems with signal amplification (receptor reserve), the response readout from compounds of different efficacies (full, partial, and possibly inverse agonist) can be quite complex due to the intermixing of the effects of partial receptor activation with those of post-receptor signal amplification. This can result in complicated connections between fractional occupancy (*f*_occup_) and fractional response (*f*_resp_) that can be fitted only by multi-parameter models. Because the present model uses different parameters for efficacy (*ε*) and amplification (*γ*), it can untangle these in a manner not possible with previous models, which intermingled these two effects within the same parameter (*τ*). Furthermore, with the present model, response data can be connected to independently measured occupancy data in a manner not possible with the operational model so that unified fit for multiple ligands can be obtained for complex cases including a) fractional responses that do not match independently measured fractional occupancies, b) responses measured after partial irreversible inactivation of the “receptor reserve” (Furchgott method), c) fractional responses that are different along distinct downstream pathways despite being initiated by the same receptor (biased agonism), and d) responses with constitutive receptor activity. We will first discuss several examples that do not involve constitutive activity (*ε*_R0_ = 0) and include a last one with *ε*_R0_ > 0.

### Response Versus Independently Measured Occupancy for Partial Agonist Series

**Figure 9:**
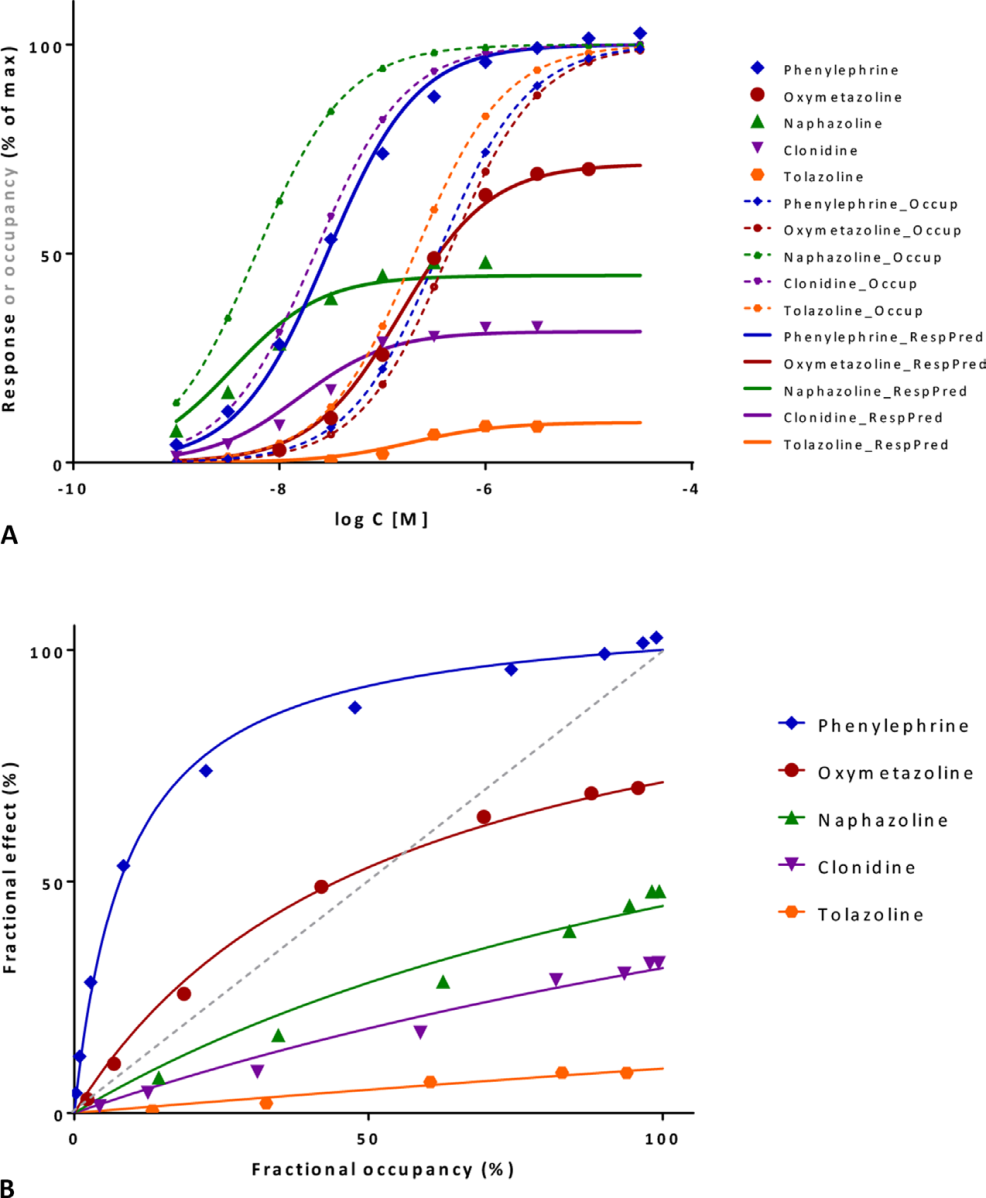
Fit of complex concentration-response data with the present model, Case I: Activity and binding data for a series of imidazoline-type α-adrenoceptor agonists (data after Ruffolo et al., 1979). **(A)** Fractional response as a function of log concentration for five compounds (symbols) fitted by the present model (Equation 4) using independently derived *K*_d_ values for binding affinity. Fitting of the response data is done by adjusting only one common *γ* (gain) and five individual *ε* (efficacy) parameters (**Table S1.C**). Fractional receptor occupancy data [calculated from the average *K*_d_ determined by two different methods (Ruffolo et al., 1979)] are also shown as dashed lines to highlight the ability of the model to account for the ligand-dependent mismatch between fractional response and occupancy. **(B)** Fractional response vs. occupancy data for these five compounds (symbols) and their corresponding fit with the present model fitted directly via the newly derived Equation 27. Note that the functional response can either exceed or lag behind the fractional occupancy data; for one compound (oxymetazoline), both occur depending on the ligand concentration.

**Figure 10:**
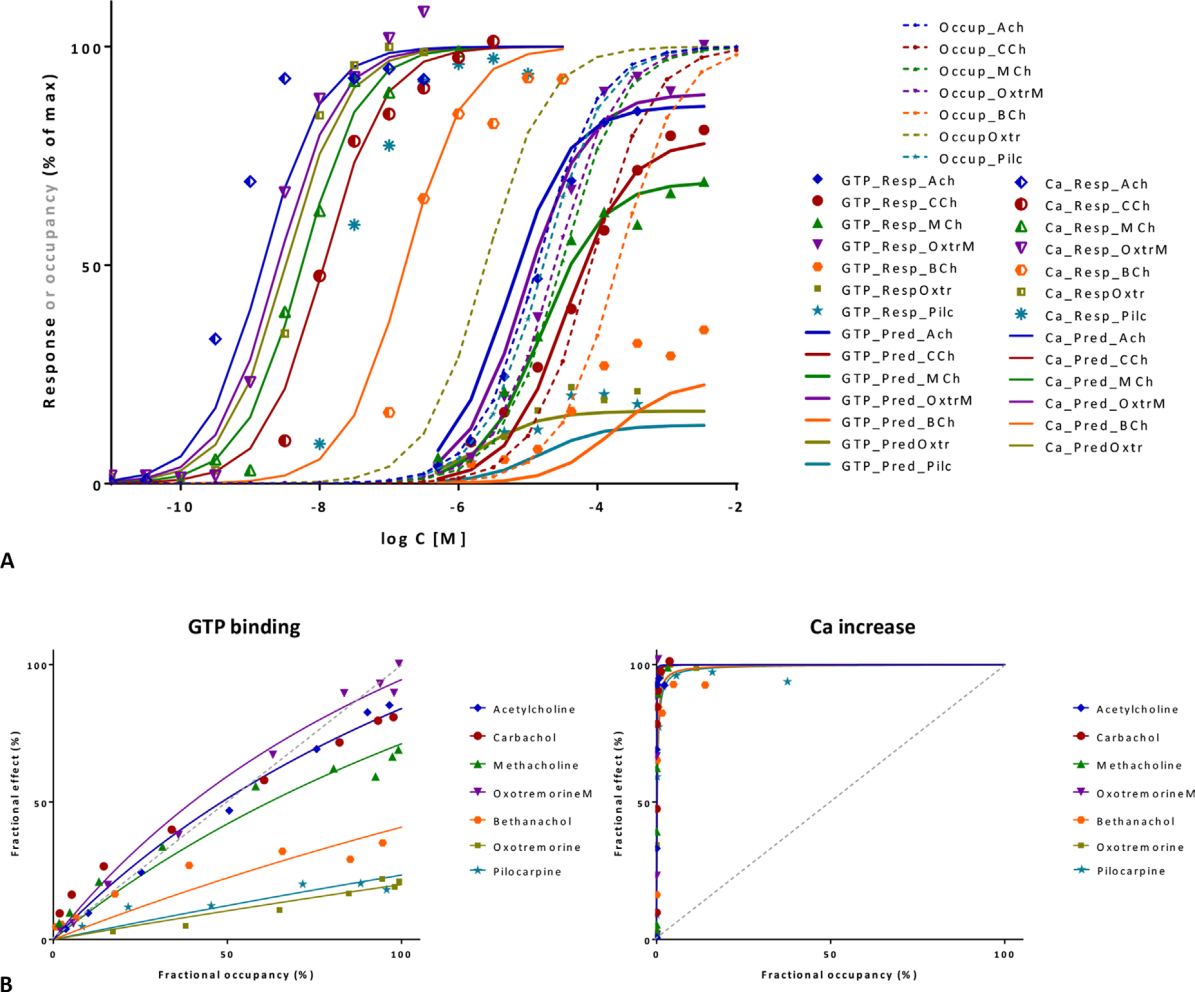
Fit of complex concentration-response data with the present model, Case II: Activity and binding data for a series of muscarinic agonists (data after Sykes et al., 2009). Responses were measured at two different points after M_3_ receptor activation: stimulation of GTP binding to Gα subunits and subsequent increase in intracellular Ca levels, respectively. **(A)** Fractional GTP and Ca responses (closed and semi-open symbols, respectively) as a function of log concentration for seven compounds fitted by the present model using independently derived *K*_d_ values for binding affinity (Sykes et al., 2009) (Equation 4; thicker and thinner lines, respectively). Fitting of the response data is done by adjusting only two common *γ* (gain) and seven individual *ε* (efficacy) parameters (**Table S2**). Fractional receptor occupancy data [calculated from the *K*_d_ determined for the receptor binding by competition assays (Sykes et al., 2009)] are also shown as dashed lines to highlight the ability of the model to account for the ligand-dependent mismatch between the two different fractional responses and occupancy. **(B)** Fractional response vs. occupancy data in the GTP (left) and Ca (right) assays for these seven compounds (symbols) and their corresponding fit with the present model fitted directly via Equation 27. The very different amplification of these two responses assessed here at two different vantage points along the pathway is quite evident from these graphs.

This involves cases where detailed response data are measured in a given system in parallel with affinity (*K*_d_) estimates for a series of compounds that include partial agonists of different efficacies, and fractional responses do not match fractional occupancies. In sufficiently complex cases, the fractional response can either exceed or lag behind the fractional receptor occupancy, sometimes even for the same compound (see **Figure 9B** for an illustration). After derivation of the corresponding equation, the ability of the present model to connect complex response data to measured occupancy will be illustrated with two sets of data involving α-adrenergic and M_3_ muscarinic receptors (**Figures 9** and **10**, respectively).

#### Linking Fractional Response to Fractional Occupancy

With the present model, one can directly link the fractional response, *f*_resp_ = *E*_/_ *_E_*_max_, to the affinity-determined fractional occupancy, *f*_occup_, which here is

(26) $$f_{occup}=\frac{\left[LR_{occup}\right]}{\left[LR_{max}\right]}=\frac{\left([LR]+[LR^{*}]\right)}{\left[R_{tot}\right]}=\frac{\left[L\right]}{[L]+K_{d}}$$

To do so (for the case of no constitutive activity), [L] in the expression of *f*_resp_ (Equation 13) is replaced with its expression as a function of *f*_occup_, from Equation 26 above, [L]=*f*_occup_ *K*_d_/(1–*f*_occup_), leading to:

(27) $$f_{resp}=\frac{\varepsilon \gamma f_{occup}}{\varepsilon (\gamma -1)f_{occup}+1}=\frac{\varepsilon \gamma }{\varepsilon (\gamma -1)}\frac{f_{occup}}{f_{occup}+\frac{1}{\varepsilon (\gamma -1)}}$$

Because of its two parameters (*ε*, *γ*), the present model allows quite flexible profiles. To avoid overparametrization-related problems, fit for a given response-system should be done using a common gain (*γ*) parameter across all ligands, and this should allow a good estimate of the overall amplification. At full occupancy (*f*_occup_ = 1), the maximum fractional response is *f*_resp,max_ = *εγ*/(*εγ* − *ε* +1), which can be less than unity for low-efficacy compounds.^3^

#### Illustration I: **α**-Adrenergic Receptor

These data involve concentration-dependent responses in a series of imidazoline type α-adrenoceptor agonists (e.g., phenylephrine, oxymetazoline, naphazoline, clonidine, and tolazoline) (Ruffolo et al., 1979) and are often used as a textbook example to illustrate mismatch between fractional receptor occupancy and response (Rang et al., 2015). Contractions of isolated rat aorta were measured as response, and receptor binding (*K*_d_) was assessed separately by two different methods. Response alone can be fitted well by a standard *E*_max_ model (Equation 10; **Supplementary Table S1.B**) or the operational model (Equation 20) giving fits of essentially identical quality (Buchwald, 2017). However, fit with the operational model results in *K* values that are different from the measured *K*_d_s and are quite meaningless for the full agonist phenylephrine (*τ* = 10^6.0^; log*K* = −1.53 vs. log*K*_d_ = −6.46) (Buchwald, 2017). While these models can be used for empirical fitting of the response data, they cannot connect the response to the independently determined binding data (*K*_d_). This can be done, however, with the present model (Equation 4) without significant loss in the quality of fit while also reducing the overall number of adjustable parameters (*n* = 6, 1 *γ* + 5 *ε*s, versus the previous *n* = 10, 5 compounds, each with a *K*_d_ and *E*_max_ or *K*_A_ and *τ*) (**Figure 9A**; **Table S1.C**). Decreasing the number of parameters is important as model simplification is always an essential consideration (George, 2000; Myung and Pitt, 2004; Buchwald, 2005; Buchwald, 2007). Hence, the present model can do more than just an empirical fit of the response data and can account for complex cases where *f*_resp_ can either exceed or lag behind *f*_occup_ depending on the ligand (**Figure 9B**). Fit here predicts a reasonable 11.9-fold amplification for this system (γ = 11.88 ± 2.02) and intrinsic efficacies ranging from 1.0 for phenylephrine to 0.009 for tolazoline (**Table S1**).

Direct fit of the *f*_resp_ vs. *f*_occup_ data (**Figure 9B**) with the newly derived Equation 27 here constrained to a single amplification *γ* parameter for all compounds also results in very good fit and essentially identical *ε* and *γ* parameters (**Table S1**). The value of the correlation coefficient, *r* ^2^ = 0.995, indicates that the model accounts for 99.5% of the variability in the quite complex response vs. occupancy data. Note that for oxymetazoline the fractional response exceeds the fractional occupancy at low occupancy (*f*_occup_<50%), but lags behind it at higher ones.

#### Illustration II: M_3_ Muscarinic Receptor

A second set of data used for illustration involves the M_3_ muscarinic receptor and a set of seven agonists including acetylcholine, carbachol, methacholine, oxotremorine, pilocarpine, and others (Sykes et al., 2009). This provides an even more complex test as two different responses were measured at consecutive vantage points after receptor activation: the stimulation of GTP binding to Gα subunits and the subsequent increase in intracellular calcium levels. Occupancy estimates are from p*K* values obtained from equilibrium competition experiments with *N*-methyl-[^3^H]scopolamine, but dynamic measurements were also performed to assess association and dissociation rate constants (*k*_on_, *k*_off_), and they resulted in somewhat shifted, but very similar p*K* values (*r* ^2^ = 0.99) (Sykes et al., 2009). If all data are placed on the same graph, it is already apparent that the amplification for the two subsequent responses quantified here is quite different: while it has to be close to unity for GTP binding (as *K*_d_s and *K*_obs_≈EC_50_s roughly overlap), it has to be around four orders of magnitude for Ca increase (as the corresponding EC_50_s are left-shifted by about four log units) (**Figure 10A**). Fit of each of the two concentration-dependent responses with the present model accounts for 98% of the variability in the data (**Figure 10B**). However, because amplification is very strong in the Ca increase assay and all agonists achieve essentially maximum activation, well-defined efficacies cannot be determined from these data alone. Therefore, a unified fit was performed on both responses using only a single set of *ε* efficacies (one each for the seven compounds) plus two *γ* gain parameters (one for each response) as adjustable parameters (**Table S2**). This confirmed that the two amplifications are indeed very different as the *γ* values obtained from this overall fit were 2.1 and 10,089 for the GTP and Ca readout, respectively. The overall fit of these two response data sets with the same set of only 9 adjustable parameters was again quite good accounting for 95% of the variability in the data (*r* ^2^ = 0.95; **Figure 10A**) with betanachol, acetylcholine, and maybe pilocarpine fitting less well. Obtained relative overall efficacies are summarized in **Table S2**. In agreement with the original observation, which looked at intrinsic activities and log *τ* values obtained from the operational model (Sykes et al., 2009), efficacies (*ε*) obtained here also correlate very well with the p*k*_off_ values (*r* ^2^ = 0.86) while having no correlation with the p*K*_d_ affinities (*r* ^2^ = 0.01). In conclusion, the present model has the potential to connect activity data assessed at different vantage points and involving different amplification steps along the same downstream pathway to affinity and intrinsic efficacy data for complex series of partial agonists.

### Response after Partial Irreversible Inactivation of the “Receptor Reserve” (Furchgott Method)

Another approach that can result in complex data difficult to fit with single unified models is the method of irreversible receptor inactivation introduced by Furchgott for the quantitative assessment of “receptor reserve” (Furchgott, 1966; Furchgott and Bursztyn, 1967). For receptors that can be irreversibly inactivated so that concentration-response functions can be established from the same preparation before and after (partial) inactivation, this approach allows the simultaneous estimation of affinity and efficacy. In the Furchgott approach, it is assumed that application of the irreversible inhibitor reduces the number of total receptors to a *q* fraction of the original, [R_tot_]’=*q*[R_tot_], thereby reducing the response-creating “stimulus” generated by a given ligand concentration [L] by the same *q* factor. Since the observed response is created via the same transduction function from the *stimulus* input, the concentrations [L] and [L]’ that create the same effect pre- and post-inhibition have to create the same *stimulus*. Assuming that the *stimulus* *S* is proportional to the concentration of occupied receptors [LR] (*S* = *ϵ*_F_[LR]) and that there is a standard hyperbolic connection between ligand concentration [L] and occupied receptors, this leads to

(28) $$[R_{tot}]\frac{\left[L\right]}{[L]+K_{d}}=q[R_{tot}]\frac{[L{]}^{\prime }}{[L{]}^{\prime }+K_{d}}$$

After some rearrangements, this results in a linear relationship between the reciprocals of equiactive concentrations that forms the basis of the Furchgott method (Furchgott, 1966; Furchgott and Bursztyn, 1967; Jenkinson, 2010):

(29) $$\frac{1}{\left[L\right]}=\frac{1-q}{qK_{d}}+\frac{1}{q}\cdot \frac{1}{[L{]}^{\prime }}$$

The slope and intercept of this line allow the simultaneous determination of *q* (fraction of receptor inactivated) and *K*_d_ (receptor affinity). The Furchgott approach makes no assumption regarding the nature of the transduction function, just that it stays the same after partial inactivation of R_tot_. Since its introduction, the method has been applied in several cases with various ligand series typically for GPCRs, such as the muscarinic acetylcholine (Furchgott and Bursztyn, 1967; Harden et al., 1986; Eglen and Whiting, 1987), opioid (Chavkin and Goldstein, 1984; Adams et al., 1990; Fox and Hentges, 2017), dopamine (Meller et al., 1987), 5-hydroxytryptamine (5-HT) (Meller et al., 1990), and A_1_-adenosine (Dennis et al., 1992; Morey et al., 1998) receptor systems.

#### Incorporation of Receptor Inactivation

Within the framework of the present model, there is a specific connection between the stimulus input, represented by the concentration of active receptors [LR*], and response as shown by Equation 12. The loss of total receptors available for ligation due to irreversible inhibition, [R_tot_]’=*q*[R_tot_], leads to a corresponding loss in the [LR*] related stimulus. From the definition of the efficacy *ε* (Equation 9), the concentration of active receptors is

(30) $$[LR^{*}]=[R_{tot}]\varepsilon \frac{\left[L\right]}{[L]+K_{d}}$$

After inactivation leaves only a *q* fraction of R_tot_, this becomes

(31) $$[LR^{*}{]}^{\prime }=q[R_{tot}]\varepsilon \frac{\left[L\right]}{[L]+K_{d}}$$

Assuming that inactivation does not affect the post-receptor signal amplification function (Equation 12), which remains the same *γ*-dependent function, and only the input stimulus is altered, the fractional response after inactivation will be

(32) $${E^{\prime }}_{/E_{max}}=\frac{\Lambda^{\prime }}{\Lambda^{\prime }+\gamma^{-1}};\Lambda^{\prime }=\frac{[LR^{*}{]}^{\prime }}{[R_{tot}]-[LR^{*}{]}^{\prime }}$$

After some transformations, this leads to:

(33) $${E^{\prime }}_{/E_{max}}=\frac{q\varepsilon \gamma [L]}{(q\varepsilon \gamma +1-q\varepsilon )[L]+K_{d}}$$

By comparing this to the expression of response before inactivation (Equation 4), it can be seen that within the framework of the present model, a *q*-fold decrease in R_tot_ translates into an apparent *q*-fold reduction of efficacy, *ε*’=*qε*. Hence, the model can be used with this formalism of “fractional efficacy” *ε*’ to fit partial inactivation data obtained in Furchgott type experiments; two examples will be discussed below.

A notable problem with the receptor reserve concept is that it was originally defined as the fraction of receptors not required to achieve maximal response (for a full agonists) (Neubig et al., 2003). However, because the transduction functions linking response to occupancy are always of asymptotic nature (i.e., they asymptotically approach a limited maximum response as ligand concentration increases), in most cases, virtually all receptors are needed for maximum response, but often, only a relatively small fraction is needed for an almost maximal response. Hence, the “reserve” or, in other words, the discrepancy between the measured *f*_resp_ and *f*_occup_ varies strongly depending on where (i.e., at what response level) it is assessed. As the half-maximal point is often used as a reference point, a frequently used way to express how much “reserve” or “spare” receptors are is to estimate the percent of receptors occupied that already produces half-maximal response (e.g., (Furchgott and Bursztyn, 1967; Kenakin and Cook, 1976; Meller et al., 1987; Adham et al., 1993; Chen et al., 1997; Morey et al., 1998; Kenakin, 2018b)). For the present model, this can be obtained from the reverse of Equation 27 linking *f*_resp_ and *f*_occup_:

(34) $$f_{occup}=\frac{f_{resp}}{\varepsilon \gamma -\varepsilon (\gamma -1)f_{resp}}$$

Hence, in a system with a gain parameter *γ*, a ligand with efficacy *ε* will produce half-maximal effect (*f*_resp_ = 0.5) at

(35) $$f_{occup}^{f_{resp}\;=\;50\%}=\frac{1}{\varepsilon (\gamma +1)}$$

For a compound that produces full activation at the receptor (*ε* = 1), half-maximal effect is produced at *f*_occup_ = 1/(1 + *γ*). This means that in a system with 10-fold amplification (*γ* = 10), half-maximal response is produced at a receptor occupancy of 9.1%, while in a system with 100-fold amplification, it is produced already at 0.99% occupancy. For agonists with lower efficacies (*ε* < 1), these values will be different even in cases where the amplification is strong enough. For ligands with low enough efficacies, half-maximal response might not even be achieved at all, as *γ* + 1 > 1/*ε* is needed to have *f*_occup_<1 in Equation 34. This interplay between efficacy and amplification can explain why the discrepancy between *f*_resp_ and *f*_occup_ can be different for different compounds even if tissue response (amplification) remains the same—a problem that confounded these studies and the notion of receptor reserve for a long time (Kenakin, 1986).

#### Illustration III: Dopamine Receptor

**Figure 11:**
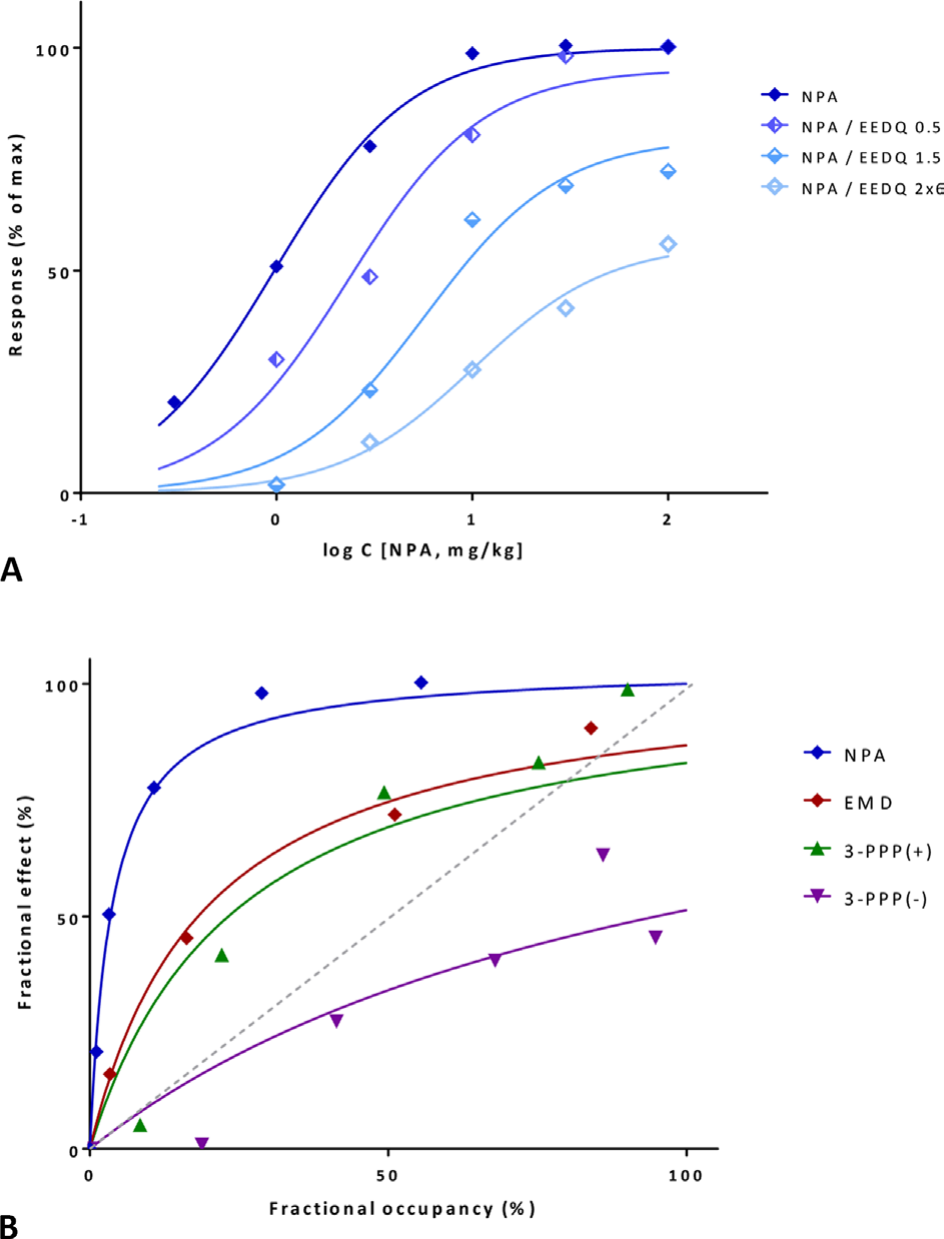
Fit of complex concentration-response data with the present model, Case III: Activity and binding data for the dopamine agonist reversal of *γ*-butyrolactone-induced striatal L-DOPA accumulation in rats (after Meller et al., 1987). **(A)** Percent of maximal response for *N*-propylnorapomorphine (NPA) and following inhibition by increasing doses of the irreversible antagonist EEDQ (0.5, 1.5, and 2×6 mg/kg). Experimental data (symbols) were fitted with the present model (lines) using Equation 33 with a single *K*_d_ and *γ* parameter and different *ε* values to account for the effect of inactivation via *q*. **(B)** Fractional response vs. occupancy data obtained in the same system (without inhibition) for four different compounds (NPA, EMD 23,448, (+)3-PPP, and (–)3-PPP). Occupancy data as calculated from *K*_d_ estimates in Meller et al. (1987). Data (symbols) were fitted with the present model (lines) using Equation 22 with a single *γ* parameter. Note that the gain parameters obtained from the same signaling pathway, but with different data sets derived from different methods are in good agreement (**Table S3**).

A first illustration is provided with a detailed data set where amplification could be estimated with two different approaches. Data are for a series of compounds acting at the dopamine receptor, which can be inhibited by the irreversible antagonist *N*-ethoxycarbonyl-2-ethoxy-1,2-dihydroquinoline (EEDQ) (Meller et al., 1987). *In vivo* dose response curves were generated in rats for the dopamine agonist reversal of *γ*-butyrolactone-induced striatal L-DOPA (L-3,4-dihydroxyphenylalanine) accumulation for four compounds: *N*-propylnorapomorphine (NPA), EMD 23,448, and (+/–)3-(3-hydroxyphenyl)-*N*-*n*-propylpiperidine [3-PPP(+) and 3-PPP(–)]. Data from one set of experiments following the Furchgott method indicates that response to NPA follows a typical hyperbolic response and is dose-dependently inhibited by EEDQ (**Figure 11A**). Fit with the present model (Equation 33, using a single *K*_d_ for NPA and a single *γ* for the response pathway) is very good (*r* ^2^=0.99) and suggests an about 43-fold signal amplification (*γ*=42.6±65.1) with EEDQ caused fractional inhibitions (*q*) estimated from the *ε* values that are in good agreement with those obtained in the original paper (Meller et al., 1987) using the classic Furchgott approach (Equation 29) (**Table S3**). The common *K*_d_ of the fit (19.0 μg/kg) also agrees well with that obtained via regression from the Furchgott approach for the highest EEDQ dose (24.4 μg/kg).

Next, the data obtained in this assay for all four compounds, but without EEDQ-mediated inhibition can also be fitted with the present model using the *f*_resp_ vs. *f*_occup_ method (Equation 27) as applied previously (**Figures 9B** and **10B**). Fractional responses plotted as a function of *f*_occup_ (with occupancies calculated from *K*_d_s values derived originally with the Furchgott method; Figure 7 in Meller et al., 1987) were fitted directly with the present model using Equation 27 with a single *γ* value. This also results in very good overall fit that accounts for as much as 95% of the overall variability in these data (*r* ^2^=0.95) (**Figure 11B**). The relative efficacies as compared to NPA as a full agonist obtained here are 0.19, 0.15, and 0.04 for EMD 23,448, 3-PPP(+), and 3-PPP(–) (**Table S3**), and they match very well those obtained originally by the Furchgott approach: 0.19, 0.12, and 0.05 (Meller et al., 1987). Finally, the two gain parameters obtained from two different data sets for this dopamine receptor system, *γ*=42.6±65.1 from the partial irreversible inhibition and *γ*=28.0±51.5 from the comparative activity of different agonists are consistent enough to support the applicability of the model here.

#### Illustration IV: Muscarinic Receptor

**Figure 12:**
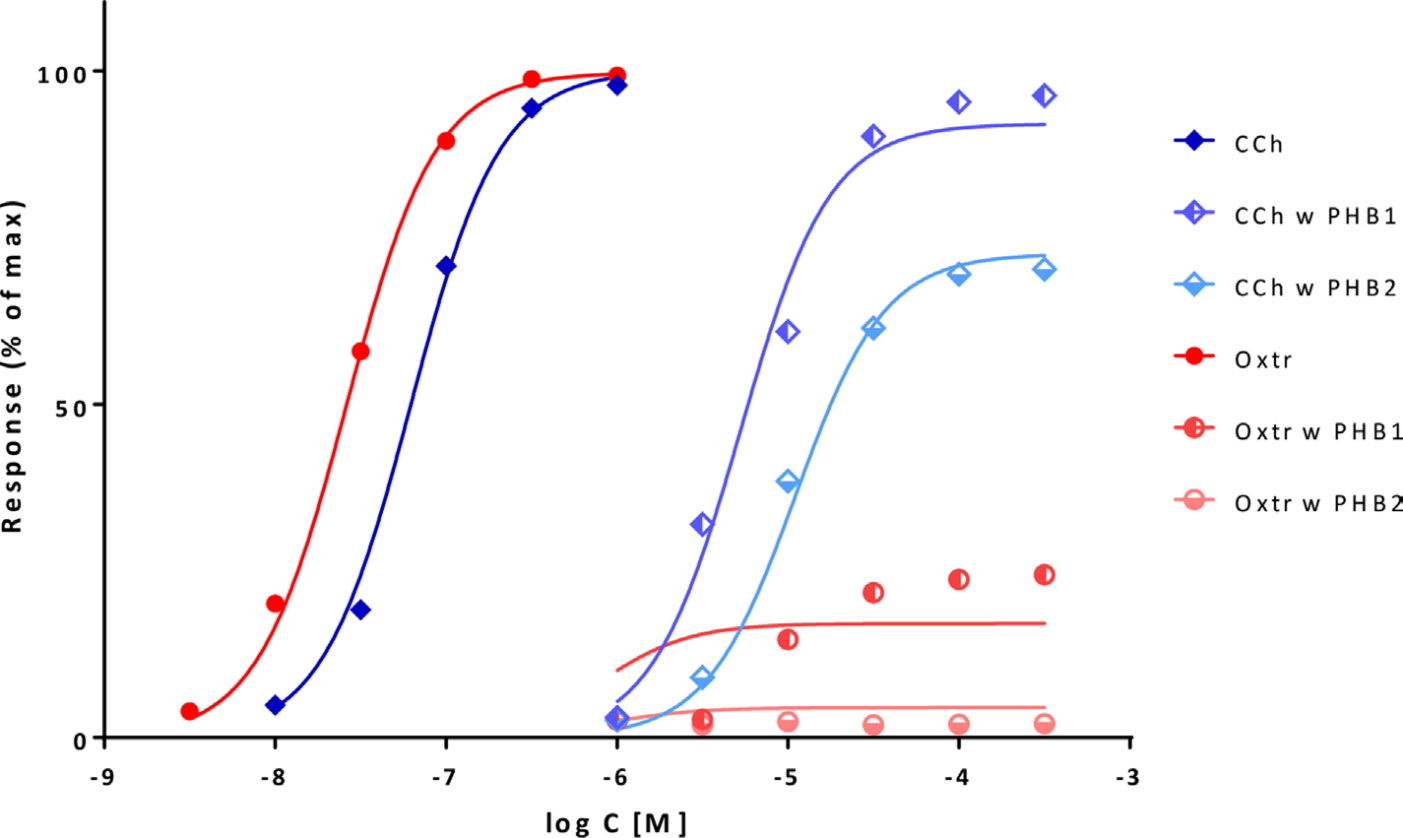
Fit of complex concentration-response data with the present model, Case IV: Fractional response curves of guinea-pig ileum contraction mediated by muscarinic receptor activation with carbachol (CCh; blue diamonds) and oxotremorine (Oxtr; red circles) in normal tissue and following inactivation through alkylation with phenoxybenzamine (PHB) at two different strengths (data after Kenakin, 1993; Kenakin and Christopoulos, 2011). Unified fit of all data with the present model is shown (lines) using a single *γ* amplification parameter for this pathway, single *K*_d_ affinity parameters for carbachol and oxotremorine, and the same *q* values for the PBA inactivation for both compounds (**Table S4**).

A second illustration is provided with an example that has been used to illustrate the capabilities of the *τ*-based operational model to explain unusual cases where full agonists become partial ones following receptor inactivation and even the order of apparent potencies changes (Kenakin, 1993; Kenakin and Christopoulos, 2011). Data are contraction of guinea-pig ileum following activation of muscarinic receptors with carbachol and oxotremorine in normal tissue and after the inactivation of muscarinic receptors by controlled alkylation with phenoxybenzamine (PHB) at two different strengths (10 μM for 10 min and 3 μM for 20 min) (Kenakin, 1993). With the present model, unified fit of all data can be achieved using a single *γ* for this pathway, single *K*_d_s for carbachol and oxotremorine, and the same *q* values for the PBA-induced inactivation for both compounds (**Figure 12**, **Table S4**). This *ε*- and *γ*-based fitting can not only account for the apparent reversal of potencies, but even results in slightly better fit than that obtainable with the operational model and with more meaningful parameters (Buchwald, 2017). Fit indicates a strong amplification (≈21,000) that is then reduced more than a thousand-fold by alkylation and a 26-fold higher affinity (log*K*_d_ of –6.04 vs. –4.62), but a 56-fold less efficacy (*ε* of 0.018 vs. 1.0) for oxotremorine as compared to carbachol (**Table S4**). These *K*_d_ estimates are in very good agreement with those obtained for guinea pig ileum in a different work by the Furchgott method (–5.83 and –4.52 for carbachol and oxotremorine, respectively) (Eglen and Whiting, 1987) or measured for the M_3_ receptor in another work (–5.61 and –4.09) (Sykes et al., 2009).

### Biased Agonism

For receptors that can signal through several downstream pathways simultaneously, such as GPCRs that are now known to couple to multiple G proteins as well as β-arrestins, it is conceivable that some ligands show differences in their ability to activate these pathways, even if they initiate from the same receptor, resulting in what has been designated as biased agonism (stimulus bias, functional selectivity, or ligand directed signaling) (Kenakin, 1995; Rajagopal et al., 2011; Kenakin and Christopoulos, 2013; Shonberg et al., 2014; Stahl et al., 2015; Ehlert, 2018; Michel and Charlton, 2018; Smith et al., 2018; Wootten et al., 2018). It is generally assumed that this could happen because such receptors can assume multiple conformational states that differ in their ability to couple to the various intracellular effectors, and ligands can show preference in stabilizing some of these conformations leading to different outcomes. However, quantifying such signaling bias is difficult, and there are increasing doubts whether it is even worthwhile for most cases (Onaran et al., 2017; Kenakin, 2018a). The most widely used quantification tools rely on ΔΔlog(*τ*/*K*) or ΔΔlog(*E*_max_/EC_50_) versus a selected reference compound (Onaran et al., 2017; Michel and Charlton, 2018). In light of the formalism of the present model, a main problem is that due to the intermix of partial agonism at the receptor followed by signal amplification, fractional responses along different pathways are nonlinearly connected even at similar efficacies, and therefore, different approaches at quantification can yield different results.

#### Quantifying Bias With the Present Model

The present model might allow a better and more clearly parametrized approach. If it can fit the data, it allows the clear separation of pathway-specific differences in amplification from those in efficacies, which then can serve as cleaner indications of bias without a need for a reference agonist. If one assumes that the amplification for each signaling pathway is the same for all ligands (i.e., *γ_k_* for pathway *k*, *k* = 1…*l*, is the same for all ligands L*_i_*, *i* = 1…*j*) then biased agonism can be suspected for ligands that show significantly different efficacies for two (or more) pathways initiating from the same receptor (i.e., *ε*_im_ vs. *ε*_in_ for the efficacy of ligand *i*, L*_i_*, for pathways *m* versus *n*). Hence, if receptor occupancy (binding affinity) data are available, one approach with the present model is to fit the data for each pathway with Equation 4 (Equation 2 if there is constitutive activity), and then compare efficacies between pathways. Bias is likely to be present for ligands with an efficacy ratio significantly different from 1. For cases where receptor occupancy data are available, the ability of the present model to connect (fractional) responses to (fractional) occupancy data (via Equation 4 or 27) provides a clear advantage, as it allows first an assessment of the adequacy of the model (i.e., whether unified gain parameters can provide adequate fit and well-defined efficacies for each response pathway) and then a detection of possible bias based on the obtained efficacy values. This is not possible with previous models, such as those based on the operational model, as they cannot incorporate experimental *K*_d_ values in their assessments. A modification of the operational model that uses experimental *K*_d_ values to constrain the regression has been proposed for bias detection by Rajagopal and co-workers (Rajagopal et al., 2011; Onaran et al., 2017). The bias quantification proposed here is in fact most similar to this one, since the *εγ* values of the present model will be very similar to the *τ* values obtained with this modified operational model, especially at large *γ* amplifications where *εγ* ≈ *εγ* − *ε* (*cf*. Equations 4 and 20). Under such conditions, comparing these *τ* ratios (Rajagopal et al., 2011; Onaran et al., 2017) is essentially the same as comparing *ε* ratios (as *γ* values are fixed for pathways). However, this operational model based approach cannot fit full agonists in systems with low amplification, where *K*_d_ and *K*_obs_ are similar (e.g., **Figure 14B and D**), because full agonism can only be achieved with large *τ’*s so that *K*_obs_ = *K*_d_/(*τ* + 1) will be shifted away from *K*_d_. This limitation is avoided with the present parametrization.

**Figure 13:**
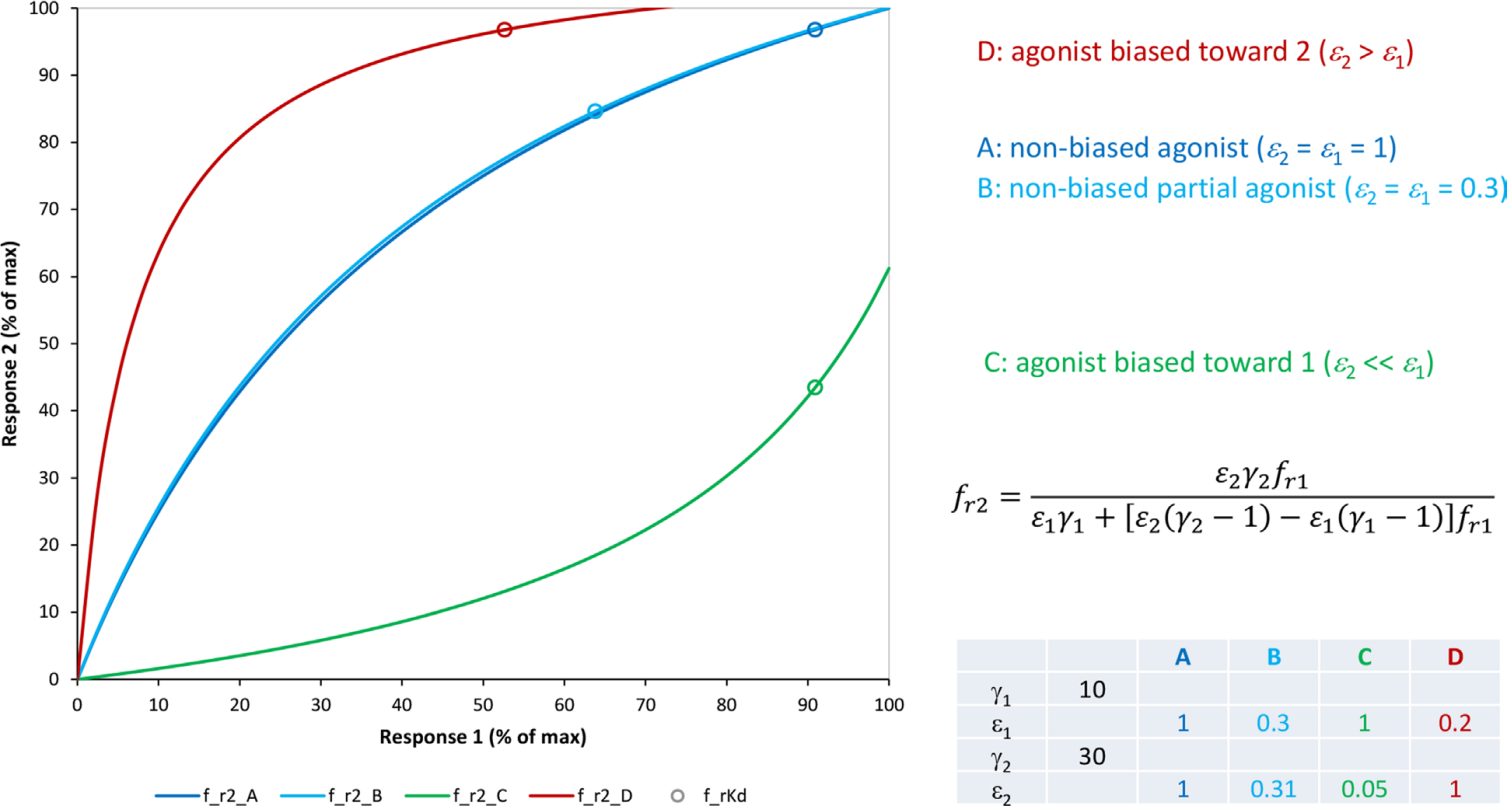
Simulated relative response (“bias”) plots with the present model (Equation 38) for two pathways with different amplifications (*γ*_1_=10 and *γ*_2_=30) and for agonists A–D with efficacies* ε*_1_ and *ε*_2_ as indicated. Small circles along the lines indicate the responses corresponding to 50% occupancy (i.e., at ligand concentrations of *K*_d_) to illustrate the mismatch between fractional responses and fractional occupancies along each pathway.

If binding data are lacking, one can use fitted *K*_d_^’^s enforcing a single set of values for all pathways. However, this way well-defined parameters are difficult to obtain, and simultaneous fit cannot be done for multiple pathways and ligands in commercial software such as GraphPad Prism. An alternative is to use the present model to directly fit the data in so-called bias plots (Gregory et al., 2010; Kenakin and Christopoulos, 2013) that are, in fact, relative response plots showing one response as a function of another one produced at the same ligand concentration (see, e.g., **Figure 13**). To derive the function directly connecting fractional responses *f*_resp1_ and *f*_resp2_ for the present model generated at the same ligand concentrations (hence, at the same fractional occupancies), *f*_occup_ will be expressed from *f*_resp_ for one of the pathways, and then used in the expression of the other. From Equation 27 (for pathway 2)

(36) $$f_{resp2}=\frac{\varepsilon_{2}\gamma_{2}f_{occup}}{\varepsilon_{2}(\gamma_{2}-1)f_{occup}+1}$$

and its reverse (Equation 34) for pathway 1

(37) $$f_{occup}=\frac{f_{resp1}}{\varepsilon_{1}\gamma_{1}-\varepsilon_{1}(\gamma_{1}-1)f_{resp1}}$$

one can link *f*_resp2_ to *f*_resp1_ directly by eliminating *f*_occup_:

(38) $$f_{resp2}=\frac{\varepsilon_{2}\gamma_{2}f_{resp1}}{\varepsilon_{1}\gamma_{1}+[\varepsilon_{2}(\gamma_{2}-1)-\varepsilon_{1}(\gamma_{1}-1)]f_{resp1}}$$

This results in various curvilinear relative response plots such as those shown in **Figure 13** generated with the parameters as indicated [intended to resemble **Figure 2** of the general review (Kenakin and Christopoulos, 2013)]. Note that for pathways that have different amplifications (*γ*_1_≠*γ*_2_), the relative response plots are still curvilinear even for ligands that do not show bias (i.e., balanced or “non-biased” agonists that have the same efficacy for both pathways, *ε*_1_=*ε*_2_):

(39) $$f_{resp2}=\frac{\gamma_{2}f_{resp1}}{\gamma_{1}+(\gamma_{2}-\gamma_{1})f_{resp1}}$$

Because of this nonlinearity, bias quantification is difficult especially for pathways that have considerably different amplifications. If the present model can fit the data, it allows the clear separation of pathway-specific differences in amplifications from those in ligand efficacies, which can then serve as indication of bias. Most biased agonism assessments published so far include only response measurements and no binding data; an example that includes affinity assessments too is discussed below for illustration. Due to the additional binding affinity data, such sets are better suited to judge the adequacy of the present model (i.e., whether unified gain parameters can provide adequate fit for each response pathway), and then detect actual bias based on the obtained efficacy values.

#### Illustration V: μ-Opioid Receptor

Biased signaling at the μ-opioid GPCR is of obvious interest for the possibility of achieving improved analgesia while reducing the unwanted side effects of opiate therapeutics, and oliceridine, a μ-opioid receptor (MOR) biased agonist, is one of the first products showing the clinical promise in developing biased agonists (Wadman, 2017). To illustrate the fitting of biased agonism data with the present model, we will use data from a recent detailed study with a series of ligands including morphine, endomorphin, met-enkephalin, DAMGO (L-tyrosyl-D-alanyl-glycyl-*N*-methyl-L-phenylalanyl-glycinol), and Pfizer standard-1 (2-(L-tyrosylamino-1-[*N*-acetly-L-phenylalanyl)-amino]-2-methylpropane hydrochloride) in cAMP and β-arrestin2 assays with the wild-type and two mutated μ-opioid receptors in which binding affinity estimates were also obtained using a [^3^H]diprenorphine competition assay (Hothersall et al., 2017).

**Figure 14:**
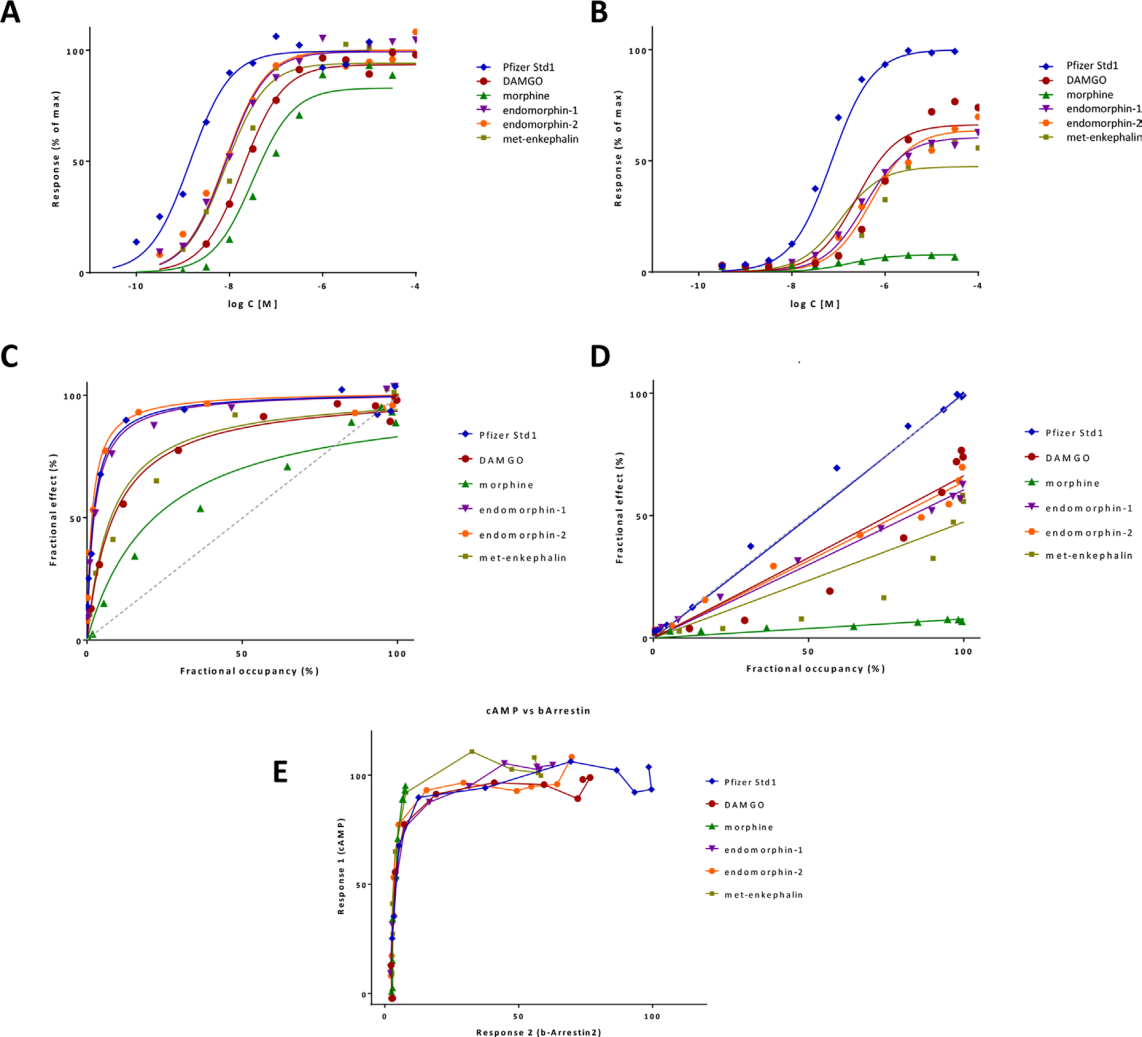
Fit of complex concentration-response data with the present model, Case V: Activity and binding data for different opioids in cells expressing wildtype μ-opioid receptor in the cAMP **(A)** and β-arrestin2 **(B)** assays, respectively (after Hothersall et al., 2017) and fitted with the present model (Equation 4). Corresponding fractional response vs. occupancy plots generated using p*K*_i_ values derived from [^3^H]diprenorphine competition assays and fitted with the model (Equation 27) are shown for cAMP **(C)** and β-arrestin2 **(D)**. A relative response plot (“bias plot”) showing the fractional responses plotted against each other is included in **(E)**.

Fit with the present model (**Figure 14A and B**) indicates that while there is essentially no amplification for the β-arrestin2 response (*γ*_2_ ≈ 1), there is a strong amplification for the cAMP response (*γ*_1_ = 62.0 ± 53.7) (**Table S5**). This is in agreement with the general observation that there is usually much stronger amplification in the G-protein mediated pathway than in the β-arrestin mediated one (Ehlert, 2018). Overall, quite good fits can be obtained accounting for 97% and 96% of the variability in the data for the cAMP and β-arrestin2 assays, respectively, even though the *K*_d_-based model struggles somewhat to fit the data for DAMGO and met-enkephalin in the β-arrestin2 assay (**Figure 14B**). Part of the problem is more evident in the corresponding fractional response versus occupancy plot (**Figure 14D**), where it is clear that the *f*_resp_ vs. *f*_occup_ plot for these two compounds has an unusual upward curvature unsuitable to fit with the present model (Equation 27). Because of the large difference in the amplifications (>60-fold), the relative response (“bias”) plot is highly curved even for balanced (non-biased) agonists (**Figure 14E**). This makes the assessment of bias difficult, as even five-fold differences in the efficacies for activation of the receptor pathways (*ε*_1_ vs *ε*_2_) can be easily overshadowed by the differences in amplifications (*γ*_1_ vs *γ*_2_). Due to the amplification in cAMP, the corresponding *ε* values cannot be well determined and have significant uncertainties when fitting directly the relative response plot with Equation 38 (**Figure 14E**); hence, without binding information (*K*_d_s), there is not enough data to determine bias in a well-defined manner. Including the binding data, and fitting with the *K*_d_-based model either directly (Equation 4; **Figure 14A and B**) or via the response vs occupancy data (Equation 27; **Figure 14C and D**) gives better defined efficacies (**Table S5**). Bias calculated this way as the ratio of the two efficacies (*ε*_cAMP_/*ε*_β-arrestin2_) ranges from 0.281 ± 0.235 for DAMGO to 1.549 ± 0.525 for endomorphin-2. Of these, only that for DAMGO, a compound sometimes used as reference (Thompson et al., 2015), can be considered as indicating significant bias as it is the only one where the ratio ±2SD does not include 1, suggesting possible bias toward β-arrestin (**Table S5**). Note, however, that because of the somewhat poorer fit of the *K*_d_-based model of the β-arrestin response for DAMGO, this conclusion should be treated with some caution. Further, in agreement with the original publication, this bias is not significant compared to Pfizer standard-1 as reference, since that compound also has a slight bias toward β-arrestin (0.783 ± 0.662).

### Constitutive Activity

All examples discussed so far involved applications of the three-parameter model (**Figure 1C**) that assumes no constitutively active receptors (i.e., no ligand-free R* form). As a simple illustration of the ability of the present general model to fit responses obtained from systems with constitutive activity, a case with opioid receptors of different constitutive activity is included below.

#### Illustration VI: μ- and **δ**-Opioid Receptors

**Figure 15:**
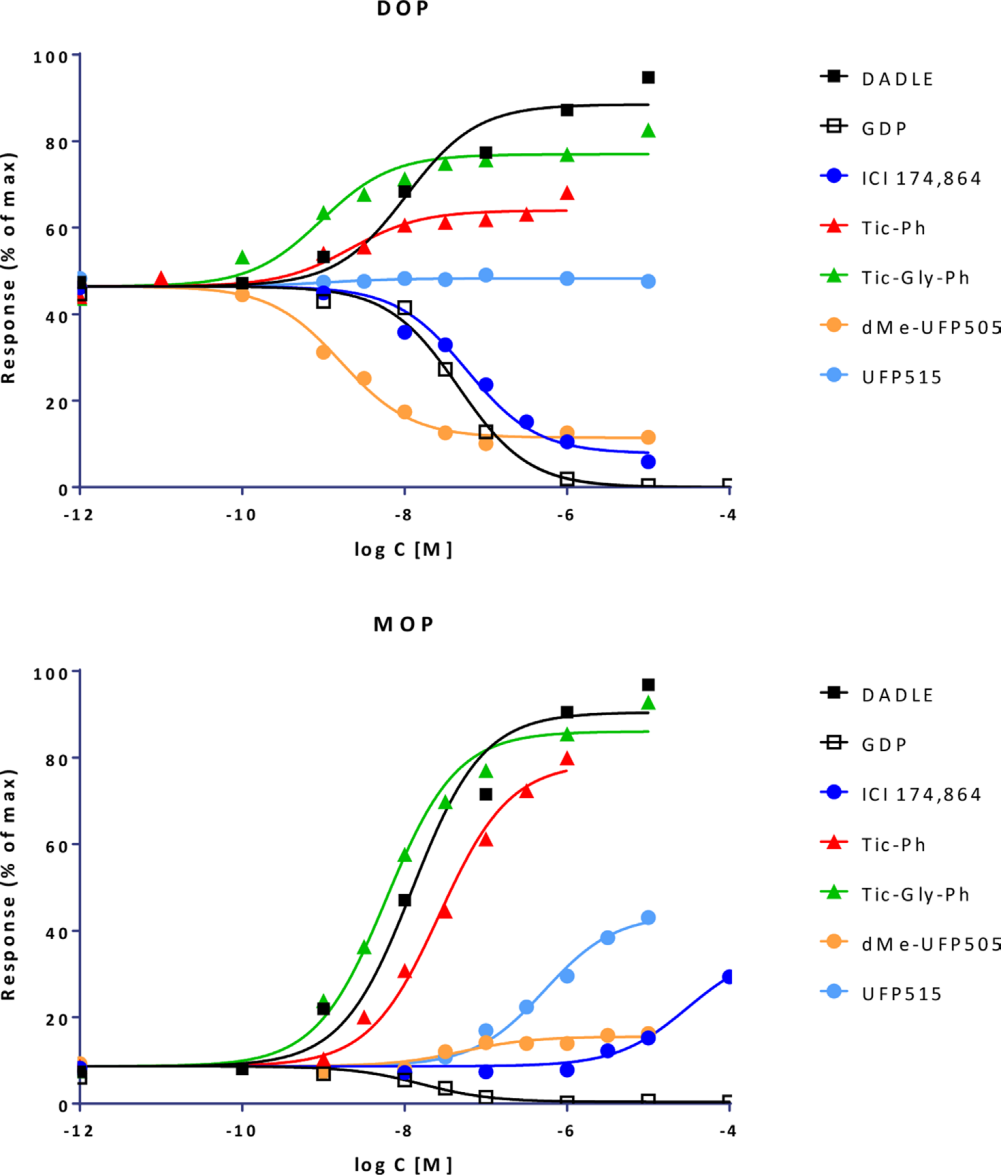
Fit of complex concentration-response data with the present model, Case VI: Activity data for fractional receptor-G protein coupling obtained with different opioids that share a peptidomimetic scaffold in **δ**- and **μ**-opioid receptors (top and bottom, respectively; after Vezzi et al., 2013) and fitted with the present model (Equation 19).

A set of fractional receptor-G protein coupling (FRC) data assessed via a cell-free bioluminescence resonance energy transfer (BRET) assay for δ- and μ-opioid receptors (DOR, MOR) with different opioid compounds that share a peptidomimetic scaffold (Vezzi et al., 2013) was fitted with the present model assuming constitutive activity, but no amplification (*ε*_R0_>0, *γ*=1; **Figure 1B**, Equation 19). This is included simply to illustrate the ability of the model as is to fit such data with non-zero baseline (**Figure 15**) and to reproduce fitting with classic three-parameter (top, bottom, ED_50_) models. In both cases, very good fits were obtained that account for more than 99% of the variability in the data (*r* ^2^=0.991) despite the wide range of ligand efficacies (**Table S6**). Furthermore, the fitted curves indeed fully overlapped with those obtained with a standard three-parameter log agonist vs. response model (*E*_max_ with adjustable bottom); they are both shown but are indistinguishable in **Figure 15**. With the present model, *ε*_R0_ values for each of the receptors were assumed to be shared values across the ligand series (as they are receptor characteristics) resulting in values of 0.464 ± 0.007 and 0.087 ± 0.008 for the δ- and μ-opioid receptors, respectively. Note that amplification may be present, but its magnitude cannot be determined from these data alone; hence, *γ*=1 was used. An illustration of the effects of different amplifications (*γ*) on the response generated by a receptor with constitutive activity with the present model (Equation 16) for a set of ligands of various efficacies (*ε*_R0_, *ε*) is included in **Figure S2**.

## Conclusion

In conclusion, a general two-state SABRE receptor model has been proposed here together with corresponding quantitative forms (**Figure 1**). By using separate parameters for binding affinity (*K*_d_), activation efficacy (*ε*), and signal gain or amplification (*γ*), it can account for complex fractional response versus occupancy data while also maintaining an intuitive nature for its parameters. Contrary to the operational model, it can be reduced back to consecutively nested simpler models, such as the Clark equation or its *E*_max_ version for partial agonists, via special cases of its parameters (e.g., *γ*=1 for no amplification or *ε*=1 for no partial agonism). This provides a straightforward bridge to such simplified cases from a full two-state receptor model as well as to their corresponding basic equations that are more suitable for fitting by nonlinear regression. The model can connect receptor response and binding data via its efficacy and gain parameters, and several complex cases where they were measured separately were included for illustration including data from partial agonist series, partial receptor inactivation (Furchgott method), biased agonism, and constitutive activity.

## Author Contributions

PB is the sole author. He developed the concept, performed the calculations and data fittings, and wrote the manuscript.

## Funding

Part of this work was supported by a grant from the National Institutes of Health National Institute of Allergy and Infectious Diseases (1R01AI101041, PI: PB).

## Conflict of Interest Statement

The author declares that the research was conducted in the absence of any commercial or financial relationships that could be construed as a potential conflict of interest.

## Footnotes

1 This overcomes a problem of the operational model, namely that the effect *E* cannot reach its actual asymptotic limit via the hyperbolic function used there, *E*/*E*_max_ = [LR*]/([LR*] + *K_τ_*), even when all receptors are active ([LR*] = [R_tot_]). The reason is that its input, [LR*], can only increase up to [R_tot_] and not to infinity so that its actual maximum is [R_tot_]/([R_tot_] + *K_τ_*) = *τ*/(*τ* + 1), which for low *τ* (high K*_τ_*) values can be only a relatively small fraction. For a graphical illustration of this, see Fig. A2 in Buchwald (2017).

2 Except for the typical notation used, the mathematical form of the operational model is identical with that of the minimal two-state (del Castillo-Katz) model (Del Castillo and Katz, 1957; Jenkinson, 2010). In Jenkinson’s description, the *τ* used here was denoted as *E* (Jenkinson, 2010).

3 Note that the operational model not only cannot accommodate experimentally determined *K*_d_s into its fitting, but if *f*_resp_ is expressed as a function of *f*_occup_ via the same method (starting from Equation 20), a simple hyperbolic function is obtained $f_{resp}^{op.mod.}=\frac{f_{occup}}{f_{occup}+\frac{1}{\tau }}$ that cannot give any insight into the response of the system as a whole (as it involves only ligand-related individual *τ* parameters).

## Supplementary Material

Data Sheet S1 A Detailed derivation of the main equation for the present model, Supplementary Tables S1–S6, and Supplementary Figures S1–S3.

Data Sheet S2 A GraphPad Prism file implementation of the present model.
