# Supplementary material for: A Receptor Model With Binding Affinity, Activation Efficacy, and Signal Amplification Parameters for Complex Fractional Response Versus Occupancy Data
Source: Front Pharmacol. 2019 Jun 11;10:605. doi: 10.3389/fphar.2019.00605 (PMC6580154; doi:10.3389/fphar.2019.00605)
Supplement: Data Sheet S1 — A Detailed derivation of the main equation for the present model, Supplementary Tables S1–S6, and Supplementary Figures S1–S3. [file DataSheet_1.pdf]

Supplementary Material

A Receptor Model with Binding Affinity, Activation Efficacy, and Signal Amplification Parameters for Complex Fractional Response versus Occupancy Data

Peter Buchwald*

*** Correspondence:** Peter Buchwald: pbuchwald@med.miami.edu

1 Appendix 1. Derivation of the main equation for the present model and of its simplified forms

To obtain the quantitative form corresponding to the full two-state model with the present formalism, we will calculate the fractional response, $f_{\mathrm{resp}} = E/E_{\mathrm{max}}$, obtained as output of the transduction (amplification) function corresponding an input that is the fraction of activated receptors, f_{act}, at a given ligand concentration [L] and use the parameter definitions to eliminate the unknown receptor concentrations. To recapitulate, in the present formalism, binding is characterized by the dissociation constant K_{d} that represents an ensemble average for all active and inactive forms:

$$K_d = \frac{[L]([R]+[R^*])}{([LR]+[LR^*])} \qquad (A1)$$

Ligand efficacy is characterized with a unitless ε parameter that represents the fraction of ligand-bound receptors that are active:

$$\varepsilon = \frac{[LR^*]}{[LR]+[LR^*]} \qquad (A2)$$

In cases where constitutive activity is present (i.e., some fraction of the receptors can be active even in when not bound to a ligand), a baseline receptor efficacy ($\varepsilon_{\mathrm{R0}}$) is defined in a similar manner (the fraction of unbound receptors that are active):

$$\varepsilon_{R_0} = \frac{[R^*]}{[R]+[R^*]} \qquad (A3)$$

With these definitions, the fraction of activated receptors f_{act}, which is proportional with the effect right after the receptor, can be expressed as a function of [L]:

$$f_{\mathrm{act}} = \frac{[R^*]+[LR^*]}{R_{tot}} = \frac{[R^*]+[LR^*]}{[R]+[R^*]+[LR]+[LR^*]} = \frac{\varepsilon_{R0}([R]+[R^*])+\varepsilon([LR]+[LR^*])}{[R]+[R^*]+[LR]+[LR^*]} = \frac{\varepsilon_{R0}([R]+[R^*])+\varepsilon\frac{[L]}{K_d}([R]+[R^*])}{([R]+[R^*])+\frac{[L]}{K_d}([R]+[R^*])} \qquad (A4)$$

Which after elimination of the receptor concentration terms results

$$f_{\text{act}} = \frac{\varepsilon_{R0}K_d + \varepsilon[L]}{[L]+K_d} \quad (A5)$$

This, after an odds-ratio type transform

$$\Lambda = \frac{f_{act}}{1-f_{act}} \quad (A6)$$

will serve as input for the present hyperbolic-type amplification function that uses $\gamma = [R_{tot}]/K_\gamma$ as its parameter:

$$E/E_{max} = f_{resp} = \frac{\Lambda}{\Lambda+\frac{1}{\gamma}} = \frac{\frac{f_{act}}{1-f_{act}}}{\frac{f_{act}}{1-f_{act}}+\frac{1}{\gamma}} = \frac{f_{act}\gamma}{f_{act}(\gamma-1)+1} \quad (A7)$$

Introducing f_{act} here and performing the corresponding algebraic transformations

$$E/E_{max} = \frac{\frac{\varepsilon_{R0}K_d+\varepsilon[L]}{[L]+K_d}\gamma}{\frac{\varepsilon_{R0}K_d+\varepsilon[L]}{[L]+K_d}(\gamma-1)+1} = \frac{\varepsilon_{R0}K_d\gamma+\varepsilon[L]\gamma}{\varepsilon_{R0}K_d\gamma+\varepsilon[L]\gamma-\varepsilon_{R0}K_d-\varepsilon[L]+K_d+[L]} \quad (A8)$$

leads to the final general form for the present full (four-parameter) model:

$$E/E_{max} = \frac{\varepsilon\gamma[L]+\varepsilon_{R0}\gamma K_d}{(\varepsilon\gamma-\varepsilon+1)[L]+(\varepsilon_{R0}\gamma-\varepsilon_{R0}+1)K_d} \quad (A9)$$

For special cases of its parameters, this general form (Figure 1A) leads to simplified forms that can be used when adequate. For example, if there is no constitutive activity (no R* form), $\varepsilon_{R0} = 0$, and this leads to the case and equation shown in Figure 1C (three parameter model as introduced earlier (Buchwald, 2017)):

$$E/E_{max} = \frac{\varepsilon\gamma[L]+0\gamma K_d}{(\varepsilon\gamma-\varepsilon+1)[L]+(0\gamma-0+1)K_d} = \frac{\varepsilon\gamma[L]}{(\varepsilon\gamma-\varepsilon+1)[L]+K_d} \quad (A10)$$

On the other hand, if there is no amplification (or there is not enough data and a model with fewer parameters is needed), $\gamma = 1$, and this leads to the model shown in Figure 1B:

$$E/E_{max} = \frac{\varepsilon 1[L]+\varepsilon_{R0}1K_d}{(\varepsilon 1-\varepsilon+1)[L]+(\varepsilon_{R0}1-\varepsilon_{R0}+1)K_d} = \frac{\varepsilon[L]+\varepsilon_{R0}K_d}{[L]+K_d} \quad (A11)$$

Further, if there is neither constitutive activity, $\varepsilon_{R0} = 0$, nor signal amplification, $\gamma = 1$, both of the above forms collapse to a form corresponding to the E_{max} model of partial agonism (Figure 1D):

$$E/E_{max} = \frac{\varepsilon[L]}{[L]+K_d} \quad (A12)$$

Finally, if there is no partial agonism (all occupied receptors are active), $\varepsilon = 1$, and this converts to the simple case of Clark equation (Figure 1F):

$$E/E_{max} = \frac{[L]}{[L]+K_d} \quad (A13)$$

2 Supplementary Tables

Supplementary Table S1. Parameters from fitting of data from Figure 9 (imidazoline-type α-adrenoceptor agonists; (Ruffolo et al., 1979)) with the present model. All fittings were done with models implemented in GraphPad Prism, and in addition to the calculated parameters (shown with their calculated standard errors) descriptors of the quality of fit (correlation coefficient, r^2, and sum of squared errors, SSE) are also included.

**Model**	**Parameter**	**Phenylephrine**	**Oxymetazoline**	**Naphazoline**	**Clonidine**	**Tolazoline**
**A**. Experimental data (from (Ruffolo et al., 1979))						
	$E_{max,L}$ ($f_{resp,max}$)	1.000	0.730	0.480	0.330	0.100
	log ED_{50}	-7.55	-6.77	-8.20	-7.60	-6.64
	log K_d[a]	-6.46	-6.36	-8.23	-7.66	-6.69
**B**. Standard E_{max} [Prism: log(agonist) vs. response - variable slope (four parameters), eq. 10; Hill slope = 1, Bottom = 0]						
	Top	99.6 ± 1.26	72.9 ± 1.04	48.0 ± 0.56	33.2 ± 0.70	10.0 ± 1.08
	$LogEC_{50}$	-7.55 ± 0.035	-6.77 ± 0.029	-8.20 ± 0.027	-7.60 ± 0.047	-6.64 ± 0.174
	r^2	0.9958	0.9978	0.9972	0.9937	0.9491
	SSE	56.25	10.50	4.26	7.52	2.95
**C**. Present [Prism implementation of present model, eq. 4 or 13; K_d from experimental]						
	ε	1	0.1739 ± 0.0274	0.0637 ± 0.0106	0.0369 ± 0.0064	0.0088 ± 0.0022
	γ	11.88 ± 2.02				
	log K_d (from exp.)[a]	-6.46	-6.36	-8.23	-7.66	-6.69
	↓[b]					
	$E_{max,L}$ ($f_{resp,max}$)	1.000	0.714	0.447	0.313	0.096
	log EC_{50} (K_{obs})	-7.53	-6.82	-8.46	-7.81	-6.73
	r^2	0.9957	0.9964	0.9508	0.9736	0.9430
	SSE	57.22	17.21	76.18	31.71	3.30
**D**. Present, f_{resp} vs. f_{occup} directly [Prism implementation of present model, eq. 27]						
	ε	1	0.1738 ± 0.0272	0.0638 ± 0.0105	0.0369 ± 0.0063	0.0089 ± 0.0022
	γ	11.88 ± 2.01				
	r^2	0.9957	0.9964	0.9523	0.9745	0.9436
	SSE	57.41	17.26	73.74	30.64	3.268

[a] Experimental data, average of log K_A (Table 3) and log K_B (Table 4) from (Ruffolo et al., 1979). [b] Derived values for the present model (using eq. 14).

Supplementary Table S2. Parameters from fitting of data from Figure 10 (muscarinic agonists assessed at two different points after M_3 receptor activation; (Sykes et al., 2009)) with the present model. Simultaneous fit of two data sets (GTP, Ca) with the same set of K_d and ε parameters across all ligands could not be done in Prism, it was done in Microsoft Excel with the Solver tool after separate prefits to establish starting values. Other details are the same as in Table S1.

**Mdl**	**Parameter**	**Acetyl-choline**	**Carbachol**	**Metha-choline**	**Oxotre-morineM**	**Bethana-chol**	**Oxotre-morine**	**Pilo-carpine**
**A**. Experimental data (from (Sykes et al., 2009))								
GTP	$E_{max,L}$ ($f_{resp,max}$)	94.44	80.56	69.44	100.00	35.42	24.31	24.31
GTP	log ED_{50}	-4.99	-4.64	-5.01	-5.59	-4.62	-5.59	-5.05
Ca	$E_{max,L}$ ($f_{resp,max}$)	103.03	110.10	101.01	100.00	96.97	101.01	94.95
Ca	log ED_{50}	-9.33	-7.98	-8.52	-8.76	-6.77	-8.47	-7.72
	log K_d[a]	-4.87	-4.09	-4.52	-4.61	-3.71	-5.61	-4.78
**B**. Present [Prism implementation of present model, eq. 4 or 13; K_d from experimental]								
	ε	0.808	0.677	0.527	1.000	0.117	0.079	0.059
	γ_{GTP}	2.14						
	γ_{Ca}	10,089						
	log K_d (exp.)[a]	-4.87	-4.09	-4.52	-4.61	-3.71	-5.61	-4.78
	↓[b]							
GTP	$E_{max,L}$ ($f_{resp,max}$)	89.99	81.77	70.41	100.00	22.00	15.46	11.90
GTP	log EC_{50}	-5.15	-4.34	-4.72	-4.94	-3.76	-5.65	-4.81
Ca	$E_{max,L}$ ($f_{resp,max}$)	100.00	100.00	99.99	100.00	99.92	99.88	99.84
Ca	log EC_{50}	-8.78	-7.92	-8.25	-8.61	-6.78	-8.51	-7.56
**C**. Present, f_{resp} vs. f_{occup} directly, GTP data only [Prism implementation of present model, eq. 27]								
GTP	ε	0.749	0.749	0.584	0.907	0.282	0.124	0.148
GTP	γ	1.76 ± 0.23						
GTP	r^2	0.989	0.958	0.942	0.987	0.752	0.922	0.7019
GTP	SSE	79.7	238.8	295.7	168.9	277.1	28.5	85.3

[a] Experimental data from (Sykes et al., 2009). [b] Derived values for the present model (using eq. 14).

Supplementary Table S3. Parameters from fitting of data from Figure 11 (dopamine receptor with and without irreversible inactivation using EEDQ; (Meller et al., 1987)) with the present model. Details are the same as in Table S1.

**NPA effect w/wo EEDQ, present model**				
Parameter	**Vehicle**	**EEDQ 0.5**	**EEDQ 1.5**	**EEDQ 2x6**
Experimental – as estimated with original Furchgott method				
q	1.000	0.380	0.170	0.058
K_d			17.0	24.4
Present [Prism implementation of present model, eq. 33] (Hill coefficient n = 1.25 ± 0.14 allowed)				
ε	1.000	0.321 ± 0.424	0.085 ± 0.121	0.029 ± 0.043
γ	42.6 ± 65.1			
K_d	19.0 (9.6 – 37.6)			
r^2	0.995	0.960	0.965	0.989
SSE	28.8	156.4	136.4	20.9
**Response vs occupancy**				
Parameter	**NPA**	**EMD**	**3-PPP(+)**	**3-PPP(-)**
Experimental – as estimated with original Furchgott method				
Rel. eff. (at 50%)	1.000	0.190	0.120	0.050
f_{occup} for 50% effect	3.8%	18%	30%	
Present, f_{resp} vs. f_{occup} directly [Prism implementation of present model, eq. 27]				
ε	1.000	0.190 ± 0.328	0.148 ± 0.256	0.036 ± 0.065
γ	28.0 ± 51.5			
f_{occup} for 50% effect	3.4%	18.1%	23.2%	94.9%
r^2	0.993	0.991	0.899	0.842
SSE	59.0	53.4	888.0	504.9

Supplementary Table S4. Parameters from fitting of data from Figure 12 (guinea-pig ileum contraction mediated by muscarinic receptor activation with carbachol and oxotremorine before and after inactivation; (Kenakin, 1993; Kenakin and Christopoulos, 2011)) with the present model. Details are the same as in Table S1.

Parameter	CCh	CCh w PHB1	CCh w PHB2	Oxtr	Oxtr w PHB1	Oxtr w PHB2
Present [Prism implementation of present model, eq. 33] (Hill coefficient n = 1.67 ± 0.12 *allowed)*						
ε	1.00	1.00	1.00	0.018 ± 0.005	0.018 ± 0.005	0.018 ± 0.005
q1		0.00055 ± 0.00033			0.00055 ± 0.00033	
q2			0.00013 ± 0.00008			0.00013 ± 0.00008
log K_d	-4.62 ± 0.008			-6.04 ± 0.008		
γ	20,962 ± 12,765					
r^2	0.996	0.983	0.992	0.998	0.335	–
SSE	32.8	125.2	36.5	13.9	342.4	27.2

Supplementary Table S5. Parameters from fitting of data from Figure 14 (opioids with wildtype μ-opioid receptor in the cAMP and β-arrestin2 assays; (Hothersall et al., 2017)) with the present model and corresponding bias estimates. Details are the same as in Table S1.

	**cAMP**	**Pfizer Std1**	**DAMGO**	**morphine**	**endomorphin-1**	**endomorphin-2**	**met-enkephalin**
**A**. Experimental data (from (Hothersall et al., 2017))							
	$E_{max,L}$ ($f_{resp,max}$)	99.9	96.77	92.89	101.58	98.50	105.90
	log ED_{50}	-8.88	-7.72	-7.24	-7.98	-8.12	-7.74
	log K_d[a]	-7.16	-6.62	-6.76	-6.44	-6.30	-6.96
**B**. Present [Prism implementation of present model, eq. 4 or 13; K_d from experimental]							
	ε	0.783 ± 0.662	0.188 ± 0.157	0.073 ± 0.062	0.704 ± 0.595	1.000 ± 0.0	0.206 ± 0.172
	ε (95% CI)	0.000 – 1.000	0.000 – 0.502	0.000 – 0.196	0.000 – 1.000	–	0.000 – 0.551
	γ	62.0 ± 53.7					
	log K_d (exp.)	-7.16	-6.62	-6.76	-6.44	-6.30	-6.96
	↓						
	$E_{max,L}$ ($f_{resp,max}$)	99.56	93.49	83.03	99.33	100.00	94.16
	log EC_{50} (K_{obs})	-8.85	-7.72	-7.50	-8.08	-8.09	-8.09
	r^2	0.976	0.990	0.963	0.987	0.977	0.944
	SSE	420.7	177.4	596.4	185.9	287.0	929.5
**C**. Present, f_{resp} vs. f_{occup} directly [Prism implementation of present model, eq. 27]							
	ε	0.784 ± 0.661	0.189 ± 0.157	0.073 ± 0.062	0.707 ± 0.597	1.000 ± 0.0	0.207 ± 0.172
	γ	61.9 ± 53.5					
	r^2	0.976	0.990	0.963	0.987	0.977	0.944
	SSE	424.0	177.8	596.5	185.5	287.6	928.9

	β-Arrestin	Pfizer Std1	DAMGO	morphine	endomorphin-1	endomorphin-2	met-enkephalin
**A**. Experimental data (from (Hothersall et al., 2017))							
	$E_{max,L}$ ($f_{resp,max}$)	100.00	77.70	7.49	58.63	63.13	59.21
	log ED_{50}	-7.32	-6.02	-6.54	-6.50	-6.30	-6.09
	log K_d[a]	-7.16	-6.62	-6.76	-6.44	-6.30	-6.96
**B**. Present [Prism implementation of present model, eq. 4 or 13; K_d from experimental]							
	ε	1.000 ± 0.0	0.670 ± 0.049	0.080 ± 0.034	0.613 ± 0.051	0.646 ± 0.051	0.482 ± 0.049
	ε (95% CI)	–	0.573 – 0.768	0.013 – 0.147	0.511 – 0.715	0.543 – 0.748	0.384 – 0.580
	γ	0.968 ± 0.149					
	log K_d (exp.)	-7.16	-6.62	-6.76	-6.44	-6.30	-6.96
	↓						
	$E_{max,L}$ ($f_{resp,max}$)	100.00	66.29	7.76	60.51	63.81	47.41
	log EC_{50} (K_{obs})	-7.15	-6.61	-6.76	-6.43	-6.29	-6.95
	r^2	0.989	0.914	0.256	0.989	0.975	0.841
	SSE	209.7	893.6	32.4	74.4	187.6	1022.0
**C**. Present, f_{resp} vs. f_{occup} directly [Prism implementation of present model, eq. 27]							
	ε	1.000 ± 0.0	0.670 ± 0.049	0.080 ± 0.034	0.613 ± 0.051	0.646 ± 0.051	0.482 ± 0.049
	γ	0.968 ± 0.149					
	r^2	0.989	0.914	0.256	0.989	0.975	0.841
	SSE	209.7	893.3	32.4	74.4	187.7	1022.0

Bias assessment:

	Bias	Pfizer Std1	DAMGO	morphine	endomorphin-1	endomorphin-2	met-enkephalin
**1. cAMP**							
	ε	0.783 ± 0.662	0.188 ± 0.157	0.073 ± 0.062	0.704 ± 0.595	1.000 ± *0.329*	0.206 ± 0.172
	ε (95% CI)	0.000 – 1.000	0.000 – 0.502	0.000 – 0.196	0.000 – 1.000	–	0.000 – 0.551
2. **β-Arrestin**							
	ε	1.000 ± *0.047*	0.670 ± 0.049	0.080 ± 0.034	0.613 ± 0.051	0.646 ± 0.051	0.482 ± 0.049
	ε (95% CI)	–	0.573 – 0.768	0.013 – 0.147	0.511 – 0.715	0.543 – 0.748	0.384 – 0.580
Ratio							
	ε_{cAMP} / $\varepsilon_{\beta Arr}$	0.783 ± 0.663	0.281 ± 0.235	0.914 ± 0.859	1.148 ± 0.976	1.549 ± 0.525	0.428 ± 0.360

Supplementary Table S6. Parameters from fitting of data from Figure 15 (opioids in δ- and μ-opioid receptors – DOP and MOP, respectively; (Vezzi et al., 2013)) with the present model. Details are the same as in Table S1.

	DOP	DADLE	GDP	ICI 174,864	Tic-Ph	Tic-Gly-Ph	dMe-UFP505	UFP515
**A**. Experimental data (from (Vezzi et al., 2013))								
	$E_{max,L}$ ($f_{resp,max}$)	1.00	–	0.07	0.70	0.84	0.12	0.48
	log ED_{50}	-7.60	–	-7.20	-8.11	-8.58	-8.46	–
**B**. Present [Prism implementation of present model, eq. 2 or 17]								
	ε_{R0}	0.464 ± 0.007						
	ε	0.885 ± 0.017	0.00 ± 0.016	0.078 ± 0.019	0.640 ± 0.014	0.770 ± 0.013	0.114 ± 0.013	0.482 ± 0.013
	log K_d	-7.98 ± 0.11	-7.34 ± 0.08	-7.24 ± 0.10	-8.66 ± 0.21	-9.03 ± 0.13	-8.78 ± 0.11	-8.98 ± 2.21
	r^2	0.954	0.991	0.980	0.915	0.941	0.992	–
	SSE	104.6	24.2	34.3	39.7	73.9	13.2	4.5

	MOP	DADLE	GDP	ICI 174,864	Tic-Ph	Tic-Gly-Ph	dMe-UFP505	UFP515
**A**. Experimental data (from (Vezzi et al., 2013))								
	$E_{max,L}$ ($f_{resp,max}$)	1.00	–	–	0.95	0.93	0.23	0.56
	log ED_{50}	-7.84	–	–	-7.52	-8.21	-7.39	-6.43
**B**. Present [Prism implementation of present model, eq. 2 or 17]								
	ε_{R0}	0.087 ± 0.008						
	ε	0.905 ± 0.020	0.004 ± 0.017	0.355 ± 0.077	0.788 ± 0.025	0.861 ± 0.018	0.155 ± 0.018	0.437 ± 0.030
	log K_d	-7.91 ± 0.07	-7.79 ± 0.55	-4.52 ± 0.38	-7.57 ± 0.07	-8.23 ± 0.06	-7.29 ± 0.65	-6.28 ± 0.15
	r^2	0.977	0.849	0.979	0.991	0.985	0.891	0.987
	SSE	199.8	8.2	8.2	48.2	102.7	9.6	16.9

3 Supplementary Figures

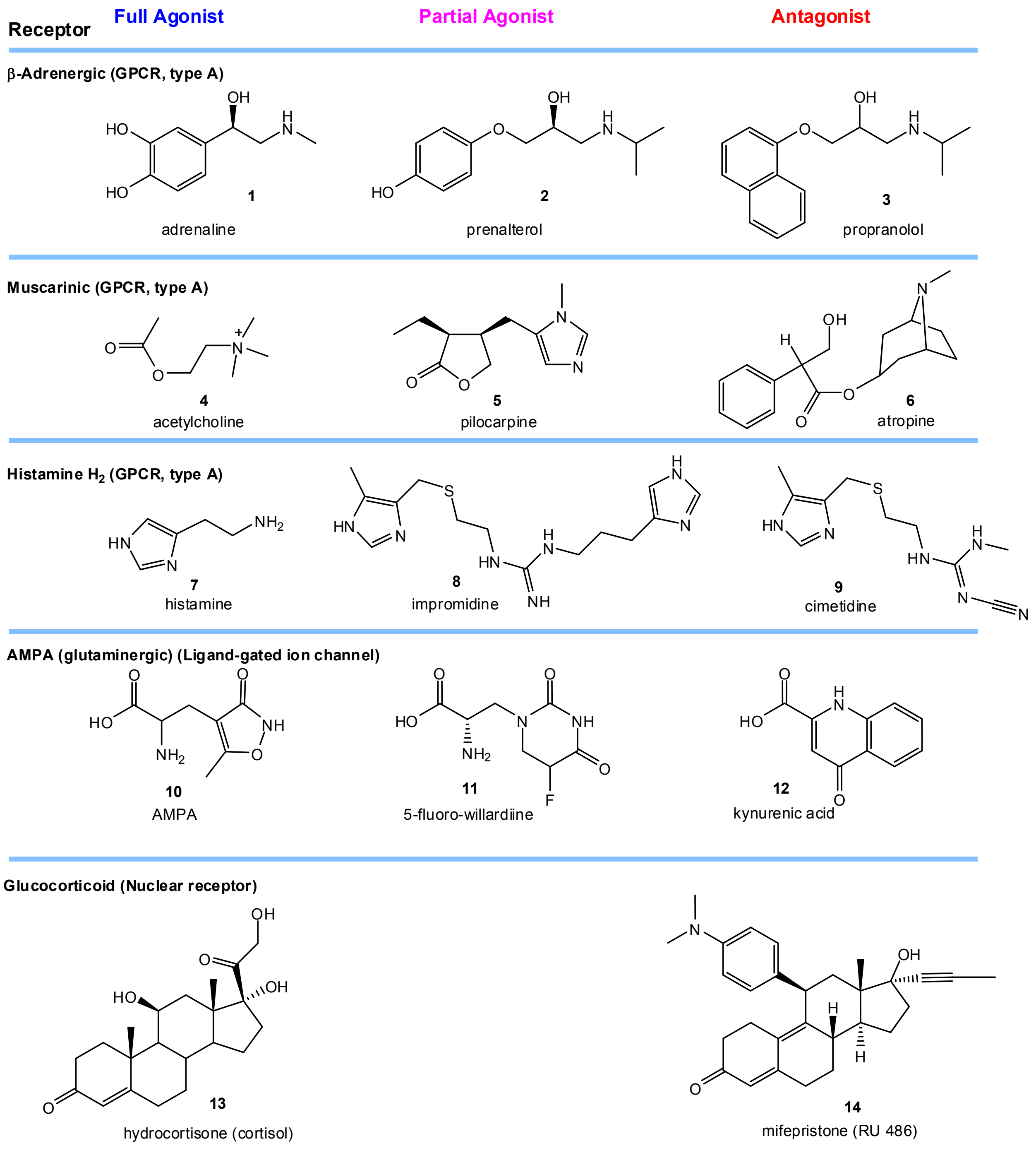

Supplementary Figure S1. Chemical structure of some representative full agonist, partial agonist, and antagonist ligands for selected receptors as indicated. While maintaining important structural elements (pharmacophores) of the full agonists, partial agonists and antagonists also tend to incorporate additional building blocks often resulting in larger overall structures.

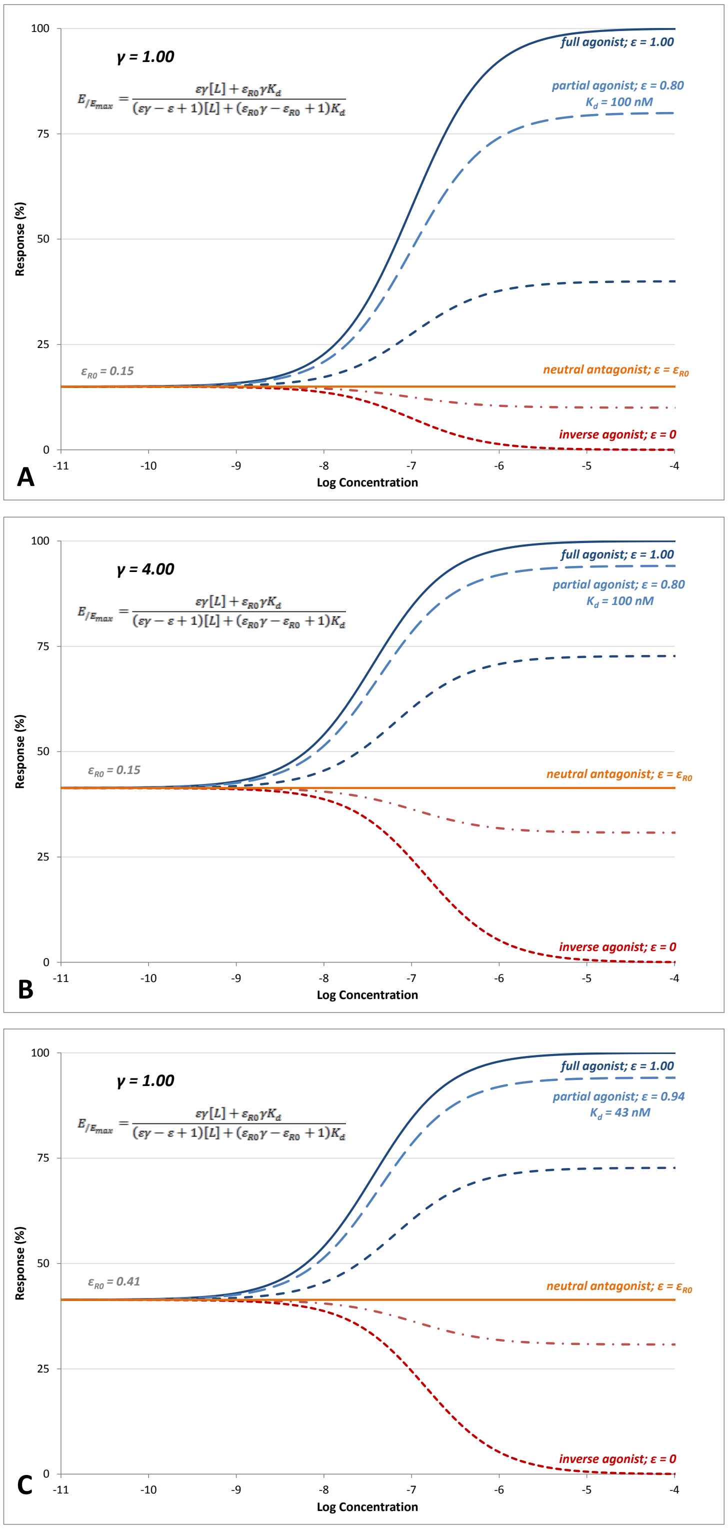

Supplementary Figure S2. Illustration of the effects of different amplification (γ) and efficacy parameters (ε_{R0}, ε) on the response of the present model (fully general form, eq. 16). Note that because of the interplay between ε and γ, the same response can be reproduced at different amplifications with a different set of εs and K_ds (B vs. C).

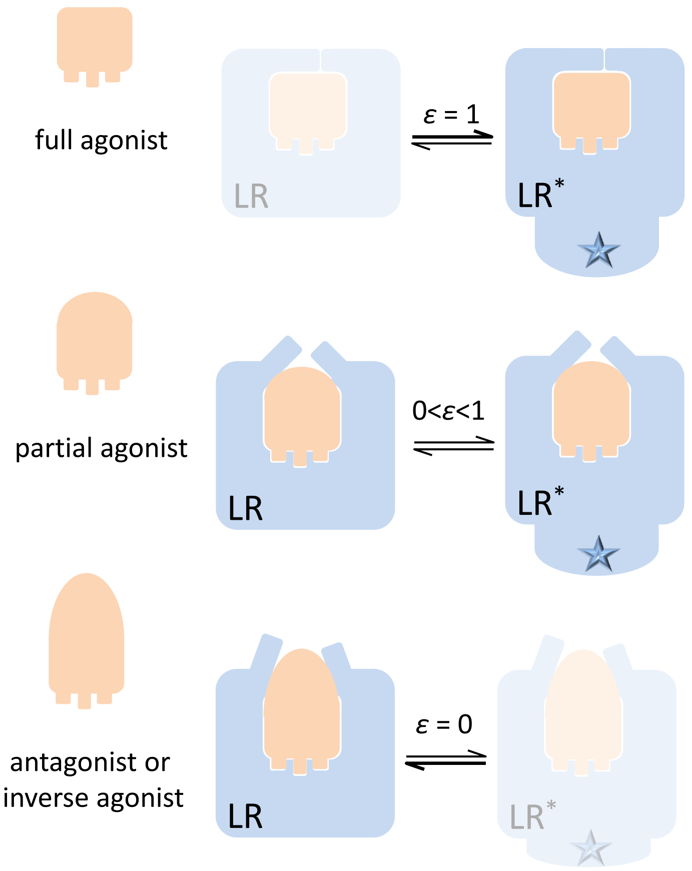

Supplementary Figure S3. A set of possible simplified mechanisms to account for the different efficacies of full and partial agonists as well as antagonists or inverse agonists in activating the receptor. The inactive and active states of the receptor are in dynamic equilibrium; upon binding, the ligand shift the position of the equilibrium. Partial agonists are not able to shift the equilibrium toward the active state as efficaciously as full agonists, a possible mechanism being that they hinder the closure of the ligand-binding domain (LBD) that makes activation more likely. If the extent (probability) of activation depends on the likelihood of the LBD to occupy its completely closed conformation, then the extent to which partial or inverse agonists hinder its closure (such as in Figure 6 or Figure 7) is also related to their efficacy in allowing activation of the receptor.
